# Optimizing genome editing strategy by primer-extension-mediated sequencing

**DOI:** 10.1038/s41421-019-0088-8

**Published:** 2019-03-26

**Authors:** Jianhang Yin, Mengzhu Liu, Yang Liu, Jinchun Wu, Tingting Gan, Weiwei Zhang, Yinghui Li, Yaxuan Zhou, Jiazhi Hu

**Affiliations:** 1 The MOE Key Laboratory of Cell Proliferation and Differentiation, Genome Editing Research Center, School of Life Sciences, Peking University, Beijing, 100871 China; 2 Peking-Tsinghua Center for Life Sciences, Peking University, Beijing, 100871 China

**Keywords:** Genome-wide association studies, Double-strand DNA breaks

## Abstract

Efficient and precise genome editing is essential for clinical applications and generating animal models, which requires engineered nucleases with high editing ability while low off-target activity. Here we present a high-throughput sequencing method, primer-extension-mediated sequencing (PEM-seq), to comprehensively assess both editing ability and specificity of engineered nucleases. We showed CRISPR/Cas9-generated breaks could lead to chromosomal translocations and large deletions by PEM-seq. We also found that Cas9 nickase possessed lower off-target activity while with some loss of target cleavage ability. However, high-fidelity Cas9 variants, including both eCas9 and the new FeCas9, could significantly reduce the Cas9 off-target activity with no obvious editing retardation. Moreover, we found AcrIIA4 inhibitor could greatly reduce the activities of Cas9, but off-target loci were not so effectively suppressed as the on-target sites. Therefore, PEM-seq fully evaluating engineered nucleases could help choose better genome editing strategy at given loci than other methods detecting only off-target activity.

## Introduction

The bacterial defense system CRISPR-Cas (clustered regularly interspaced short palindromic repeats and CRISPR-associated proteins) has been engineered to be a versatile genome editing tool^1–6^. CRISPR/Cas9 consists of a guide RNA (gRNA) complementary to target genomic sequence and a Cas9 nuclease to generate a double-stranded DNA break (DSB). Besides 20-bp gRNA-complementary sequence, CRISPR/Cas9 requires extra universal nucleotides NGG adjacent to the target site, termed as protospacer adjacent motif (PAM), to initiate DNA editing, which limits the choice of targeting site but helps to improve the specificity^7^.

CRISPR/Cas9 shows great potential in genome editing, however, its off-target activity usually causes damage at imperfectly matched genomic loci or leads to chromosomal rearrangements^8–12^, limiting its application for therapeutic purpose^13^. Many efforts have been made to reduce off-target activity of CRISPR/Cas9 and high-fidelity Cas9 variants have been generated for this purpose. Cas9 D10A nickase exhibits less detectable off-target activity, but it requires two neighbor gRNA-targeting sites to initiate genome editing^9,14^. Enhanced Cas9 (eCas9) has showed lower off-target activity due to less nonspecific contacts between Cas9 and target DNA^15^. Tunable system that controls the duration time of activated Cas9 may also help to lessen undesirable damages to the genome, such as the AcrIIA4 inhibitor that blocks Cas9 activity after cleavage^16^.

Couple high-throughput sequencing methods designed for detecting DSBs were adapted to identify CRISPR/Cas9 off-target hotspots^17^. Linear amplification-mediated high-throughput genome-wide translocation sequencing (LAM-HTGTS) employs a “bait” DSB site to capture genome-wide DSBs that form translocation with it^9^. GUIDE-seq^10^ and IDLV^18^ introduce a designed DNA fragment to randomly integrate into DSB sites as cloning primer anchoring site. Digenome-seq^19^, CIRCLE-seq^20^, and SITE-seq^21^ utilize in vitro Cas9 digestion and then capture the broken ends of Cas9-induced DSBs either by deep sequencing or end-tagging strategy. Compared to in vivo DSB-mapping methods, in vitro methods show higher sensitivity but higher background, and require further in vivo verification. In this context, BLISS^22^ employs ex vivo end-tagging in crosslinked cells that may help to reduce the background with a trade-off of lower end-tagging efficiency. However, neither of the above-mentioned methods is capable of determining in vivo editing efficiency of CRISPR/Cas enzymes. In this regard, targeted sequencing is an alternative high-throughput sequencing way to roughly determine the editing efficiency of CRISPR/Cas9 through counting minor mutations generated at the Cas9 cleavage sites^2^, but PCR amplification bias leads to quantification inaccuracy. Different with targeted deep sequencing, tracking of indels by decomposition (TIDE)^23^ utilizes Sanger sequencing and a specific algorithm to evaluate insertions/deletions (indels) amplified by PCR. Both T7 endonuclease I (T7EI) assay^3^ and restriction fragment length polymorphism (RFLP)^24^ amplify indels via PCR to omit deep sequencing, but T7EI tends to miss tiny indels and RFLP relies on an appropriate restriction enzyme cutting site spanning the Cas9 target site.

To simultaneously determine editing efficiency and specificity of CRISPR/Cas9, we developed the primer-extension-mediated sequencing (PEM-seq). PEM-seq combined LAM-HTGTS with targeted sequencing and thus could sensitively detect CRISPR/Cas9 off-target sites through translocation capture and assessed their editing efficiency by quantifying imperfect Cas9-induced DSB repair products. We characterized off-target sites as well as other abnormal chromosomal structures including small indels, large deletions, and genome-wide translocations of Cas9 by PEM-seq. We also employed PEM-seq to test several widely used methods developed to reduce Cas9 off-target activity and found that the ability of PEM-seq to comprehensively assess both editing efficiency and specificity of designed CRISPR/Cas9 could greatly help choose appropriate genome editing strategy at given loci. Notably, we generated a new high-fidelity variant named further eCas9 (FeCas9) that has extremely low off-target activity with no obvious loss of editing ability compared with wild-type (WT) Cas9.

## Results

### PEM-seq sensitively identifies off-target hotspots of CRISPR/Cas9

**Fig. 1:**
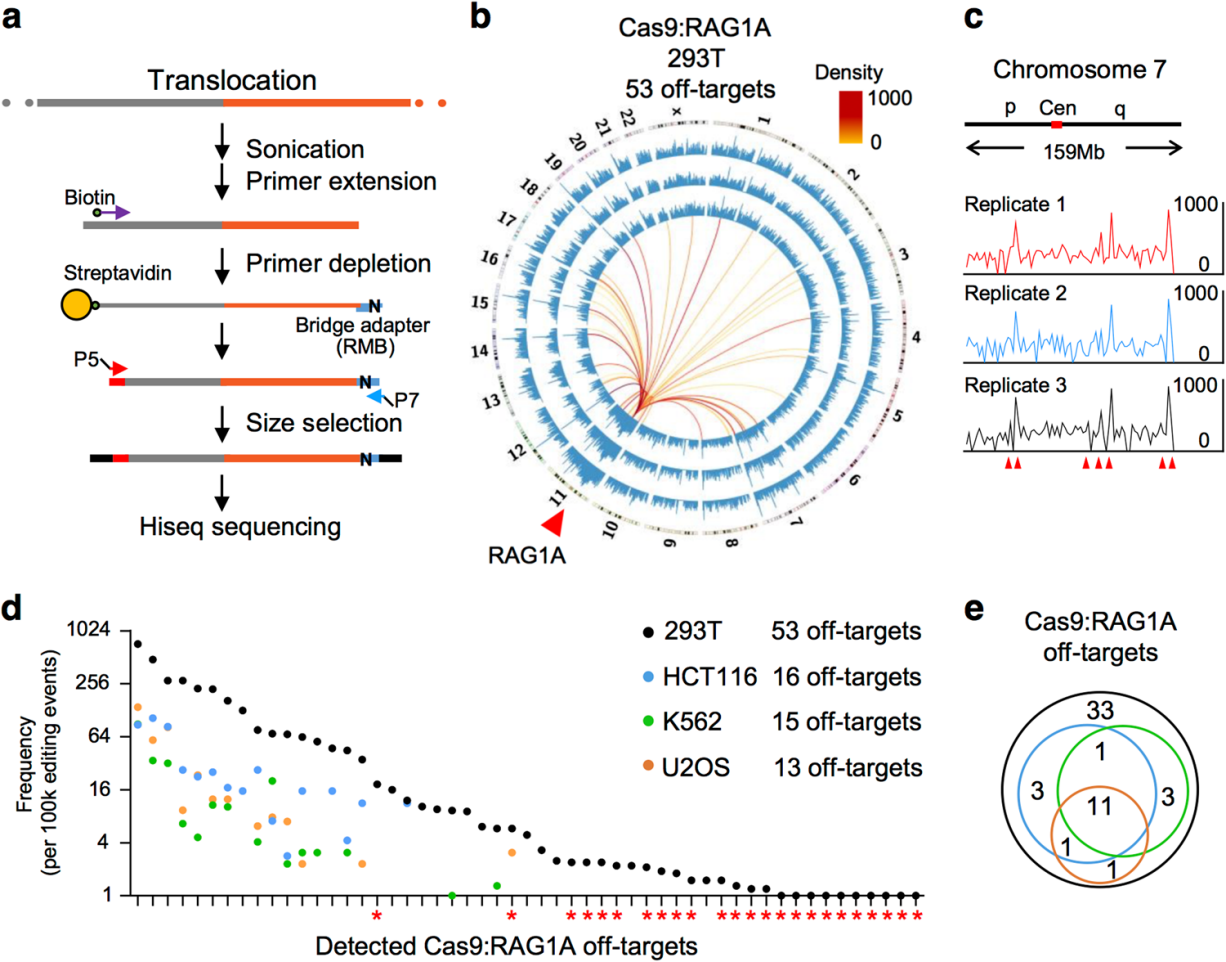
Primer-extension-mediated sequencing (PEM-seq) detects off-target hotspots of CRISPR/Cas9. **a** Overview of PEM-seq. To prepare PEM-seq libraries, primer extension generated a copy of template with biotinylated primer followed by bridge adapter ligation and DNA amplification. Gray bars represented bait region while orange ones for captured prey region; “N” indicated random molecular barcode (RMB) in the bridge adapter. Arrows indicated positions and orientations of primers. See supplemental protocols for details. **b** Circos plots of Cas9:RAG1A libraries. Three biological replicates were showed from outside to inside, and the displayed translocation junctions were 19,494, 16,005, and 18,078. Genome-wide translocation junctions binned into 5-Mb regions (blue lines) were plotted on a log scale. Chromosomes were showed with centromere to telomere in clockwise direction. Red arrow indicated the Cas9:RAG1A cleavage site. Colored lines connected the on-target site to the off-target hotspots. **c** Zoomed-in view of Cas9:RAG1A translocation junctions (binned into 2 Mb) on chromosome 7. Red arrows indicated identified off-target hotspots. Cen represented centromere and p/q indicated the chromosome arms. The Pearson correlation coefficient was 0.99 between replicates 1 and 2, 0.99 between replicates 1 and 3, 0.98 between replicates 2 and 3. **d** Scatter plot of Cas9:RAG1A off-target hotspots in 293T, HCT116, K562, and U2OS cells. *y* Axis showed frequency of each hotspot per 100,000 editing events (indels plus translocation). Red asterisks indicated the off-target hotspots detected by PEM-seq but not by linear amplification-mediated high-throughput genome-wide translocation sequencing. **e** Venn Diagram showing the overlap for RAG1A off-target hotspots among indicated cell lines. Legend is the same as showed in **d**

Translocation requires the joining of two separate DSBs, so placing a locus-specific primer at induced DSBs helps to identify other unknown DSBs, as showed by LAM-HTGTS^17^. LAM-HTGTS identifies CRISPR/Cas9 off-target sites via mapping genome-wide translocation with target cleavage site^9^. It initiates with an 80-cycle linear amplification to generate multiple copies of the original DNA fragments, which makes it difficult to distinguish PCR duplicates from the original templates. To overcome this problem and thus to fully assess CRISPR/Cas9, we developed PEM-seq. PEM-seq captures Cas9-induced-DSB outcomes including insertions, deletions, and genomic rearrangements via translocation capture as LAM-HTGTS does. To enable PEM-seq to quantify these outcomes, we conduct primer extension to generate only one copy of the original templates and then isolate these surrogate fragments to join to adapters containing a 14-bp random molecular barcode (RMB) to label each fragment (Fig. 1a). A new bioinformatic pipeline was also prepared to effectively distinguish different DSB outcomes for PEM-seq (Supplementary Fig. S1a).

In order to compare with LAM-HTGTS, we used Cas9 to target the RAG1A site with 33 off-target sites identified by LAM-HTGTS in the HEK293T cells^9^. About 20 µg CRISPR/Cas9-treated genomic DNA was used for each PEM-seq library and the bait primer was placed within 200 bp from the cleavage site (Supplementary Table S1). We generated three biological replicates for each treatment and combined them for translocation junctional hotspots analysis (Fig. 1b, c; see Materials and methods for details). Via PEM-seq, we identified total 53 off-target sites containing 24 new sites and losing 4 weak sites identified by LAM-HTGTS (Fig. 1b–d and Supplementary Table S2). To verify these off-target sites, we amplified 8 of them, including 4 new sites and 4 overlapped ones, together with RAG1A on-target sites and applied them for in vitro CRISPR/Cas9 digestion. After 20-h incubation, the uncleaved bands of RAG1A on-target site were almost gone, while the “cold” DNA fragment containing no RAG1A target sites showed undetectable cleavage (Supplementary Fig. S1b). Cas9-induced specific cleavage on the 8 selected sites varied from 18 to 60%, much lower than that of RAG1A on-target site (Supplementary Fig. S1b). In addition, we performed PEM-seq analysis on two weakest off-target sites (OT6 and OT8 as indicated in Supplementary Fig. S1b) from the same Cas9:RAG1A-targeted DNA and found a few translocation junctions between these OT6/8 with several other off-target sites (Supplementary Fig. S1c). The miss of RAG1A on-target-involved translocation in libraries from OT6/8 target baits may due to delayed cleavage timing for off-target sites compared to on-target sites^25–27^. Taken together, detected off-target sites were indeed cleaved in vivo and these data suggested a higher sensitivity of PEM-seq to detect off-target sites than LAM-HTGTS did.

To test the compatibility of PEM-seq, we applied PEM-seq to study Cas9:RAG1A off-target hotspots in other cell types including HCT116, K562, and U2OS. To test whether PEM-seq works consistently in these cell lines, we checked the general patterns of genome-wide translocation junctions of these libraries. About half of the translocation junctions located in genic region in all the cell lines, in line with previous reports^28,29^, and the distribution profiles of translocation junctions at each chromosome were similar among different cell lines (Supplementary Fig. S1d–f). Moreover, PEM-seq detected 13–16 off-target sites (Supplementary Table S2) in the three tested cell lines, all of which had occurred in the HEK293T libraries (Fig. 1d, e). These results indicated that PEM-seq can be easily adapted to detect off-target loci in any editable cell line.

### PEM-seq assesses editing ability of CRISPR/Cas9

**Fig. 2:**
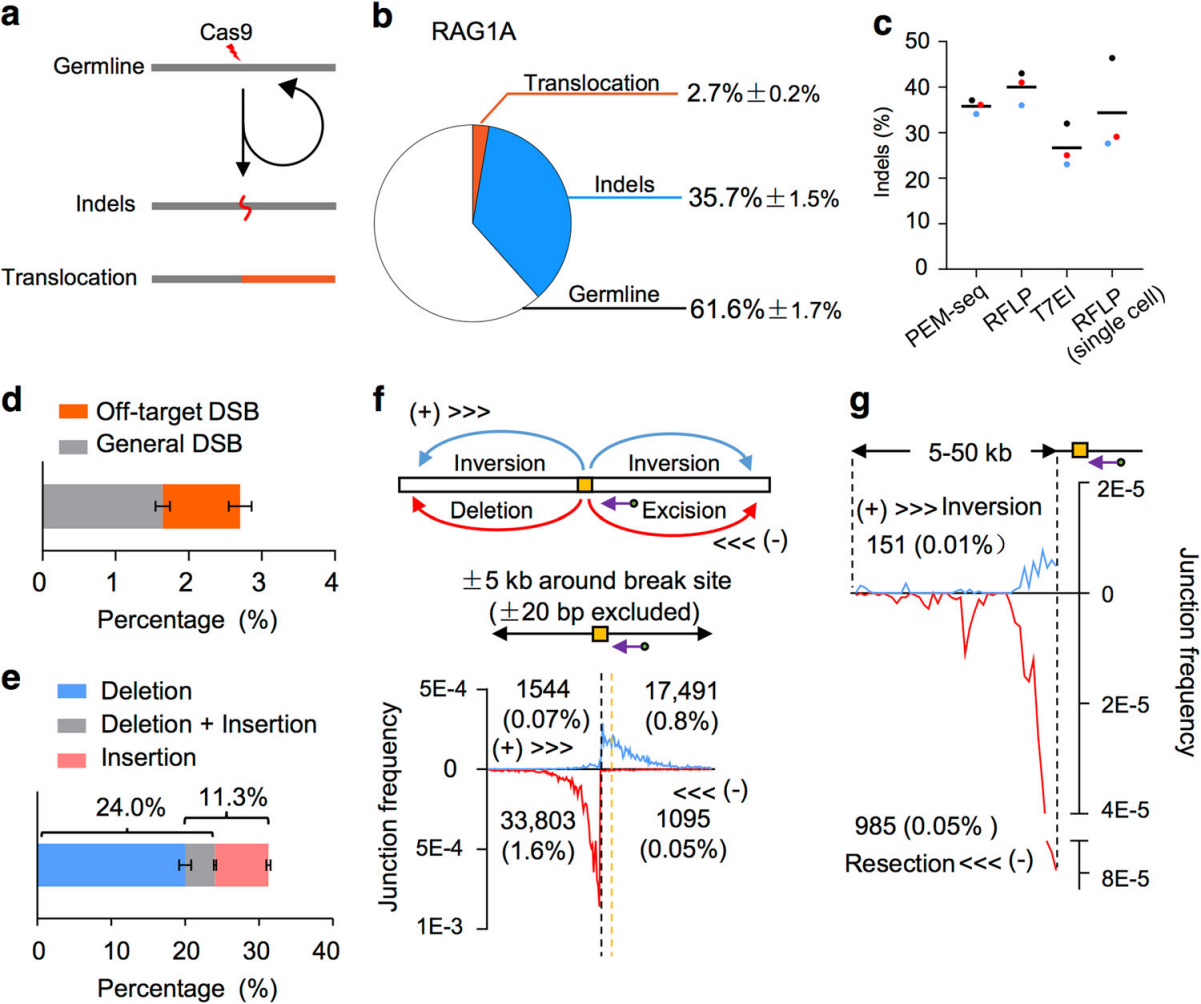
Assessing editing efficiencies of CRISPR/Cas9 by primer-extension-mediated sequencing (PEM-seq). **a** Outcomes of Cas9-induced double-strand breaks (DSBs). Germline represents uncut or perfect rejoining; indels is derived from errored rejoining; translocation involves a second DSB. **b** Percentages of germline, indels, and translocation showed by pie graph for Cas9:RAG1A. Mean ± SD. **c** Frequency of indels for Cas9:RAG1A detected by PEM-seq, restriction fragment length polymorphism (RFLP), T7EI assay, and single-cell RFLP. Averages were indicated by black lines. DNA from different batches were marked in different colors. **d** Composition of Cas9:RAG1A translocation either joined with off-target DSBs or genome-wide low-level DSBs. Mean ± SD. **e** Composition of indels of Cas9:RAG1A libraries within ±20 bp around cleavage site. Note that part of deletions also involved insertion, indicated in gray. Mean ± SD. **f** Frequency of junctions (binned into 50 bp) within ±5 kb (±20 bp excluded) around Cas9:RAG1A cleavage site. Upper schematic showed different DNA repair products detected by PEM-seq, including inversion, deletion, and excision. Yellow box indicates the Cas9 target site. Blue and red arrows indicate the translocation orientations. Purple arrow indicates the position and orientation of primer used for PEM-seq. In the bottom panel, black dotted line indicates the Cas9 cleavage site, while yellow dotted line indicates the primer locus. Junction numbers for each region were indicated. **g** Frequency of junctions (binned into 1 kb) for 5–50 kb downstream of Cas9:RAG1A cleavage site. Black dotted lines indicate the boundaries of the 5–50 kb region. Purple arrow indicates the primer used for PEM-seq. Junction numbers for each region were indicated

We next used PEM-seq to quantify editing events including translocation and indels in addition to germline of RAG1A libraries. Germline could be either uncleaved or perfectly repaired target fragments, while indels indicated insertions or deletions resulting from rejoining of the Cas9-generated broken ends (Fig. 2a). As DSB repair products, the levels of translocation and indels are proportional to the cleavage ability of nucleases^30^, so we can estimate the editing efficiency of CRISPR/Cas9 through the numbers of translocation and indels generated during gene editing. In HEK293T cells treated with Cas9:RAG1A, the levels of translocation and indels were 2.7% and 35.7%, respectively, which added up to an editing efficiency at about 38.4% (Fig. 2a and Supplementary Table S1). Even though we took titrated amounts of sequenced raw reads to perform PEM-seq analysis for RAG1A site, the percentage of indels remained consistent (Supplementary Fig. S2a). Moreover, we mixed different ratios of untreated DNA and Cas9:RAG1A-treated DNA to prepare libraries and found the percentage of indels was proportional to the input ratio (Supplementary Fig. S2b). We also employed RFLP, T7EI, and single-cell RFLP to analyze CRISPR/Cas9 at RAG1A site, and the indels were 27–40% (Fig. 2c and Supplementary Fig. S2c–e), in the same range as PEM-seq. In addition, we employed TIDE to analyze indels of Cas9:RAG1A-treated samples and found a similar level of indel percentages as PEM-seq (Supplementary Fig. S2f). These data suggested that PEM-seq could reliably quantify massive junctions accumulated at on-target sites and thus help to assess editing efficiency of CRISPR/Cas9.

### PEM-seq detects large deletions and translocation induced by CRISPR/Cas9

Translocation and indels might threaten genome stability of CRISPR/Cas9-treated cells, therefore we further analyzed the composition of identified translocation and indels after CRISPR/Cas9 treatment. About 1.1% identified translocation events occurred between target sites and off-target sites, while the other 1.6% happened between target sites and genome-wide low-level DSBs (Fig. 2d). With regards to indels, vast majority of them occurred within 20 bp around the target site, with 11.3% small insertions and 24.0% tiny deletions (Fig. 2e). Larger deletions frequently occurred within approximately 3 kb downstream of the primer with a moderate extension to 5 kb; inverted joinings also distributed in the upstream 5 kb region as previously reported^9^. In the Cas9:RAG1A-treated cells, about 2.5% of total editing events led to deletion or inversion within ±5 kb around the target site (Fig. 2f). Notably, there were low level of enriched junctions expanded as long as 50 kb from the target site downstream of the primer distributing in a biased orientation, which was a typical end resection-induced pattern. The levels of resection in the 5–50 kb region were about 0.05% of total editing events (Fig. 2g). These results indicated that CRISPR/Cas9 editing accumulates abnormal DSB repair products around the target site and PEM-seq could detect them accurately.

### Cas9 nickase shows lower off-target activity with loss of target editing efficiency

**Fig. 3:**
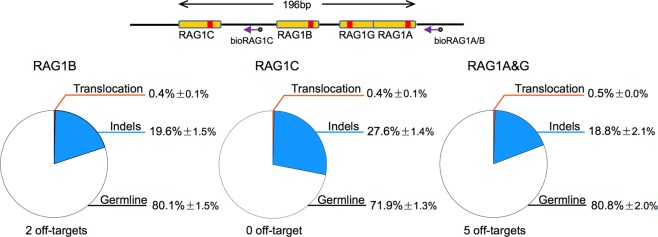
Cas9 nickase shows lower off-target activity with loss of target editing efficiency. Schematic of RAG1A, RAG1B, RAG1C, and RAG1G target site positions. Purple arrows indicated the biotinylated primers used for primer-extension-mediated sequencing. Yellow boxes represented guide RNA target sites and red bars indicated Cas9 cleavage site. In the lower panels, pie graph showed the compositions of germline, indels, and translocation for indicated Cas9 treatment in HEK293T cells. Mean ± SD

Designing several Cas9-targeting sites for a certain locus is usually used to screen for a CRISPR/Cas9 balanced for target editing ability and off-target activity. With this regard, we tested Cas9 at alternative sites RAG1B and RAG1C within a 196-bp region around RAG1A^9^. Both Cas9:RAG1B and Cas9:RAG1C showed lower editing efficiencies (20.0 and 28.0%, Supplementary Table S1) but also less off-target sites (2 and 0, Supplementary Table S3) and lower levels of genome-wide translocations (0.4% for both) (Fig. 3). Compared to Cas9:RAG1A, Cas9:RAG1C is more balanced considering the off-target activity and could be a good choice for *RAG1* gene targeting in this region, while Cas9:RAG1B is not a good choice.

Alternatively, employing Cas9 nickase to generating two neighbor DNA nicks can also be used for genome editing, and which was reported to induce less off-target damage^14^. We designed a new target site RAG1G with a 29-bp spacer between RAG1A cleavage site, and conducted *RAG1* gene targeting with RAG1A/G nickases^9^. We found RAG1A/G nickases showed an editing efficiency at 19.3% (Fig. 3 and Supplementary Table S1), only about half of that of Cas9:RAG1A (38.4%). In addition, we captured five off-target sites by PEM-seq (Fig. 3), none of which was reported by LAM-HTGTS^9^. All the five identified off-target hotspots ranked top in the RAG1A off-target list and none related to RAG1G site (Supplementary Table S3). Therefore, Cas9 nickase reduces off-target damage during genome editing with a sacrifice of editing efficiency.

### High-fidelity Cas9 variant FeCas9 showed very low off-target activity

**Fig. 4:**
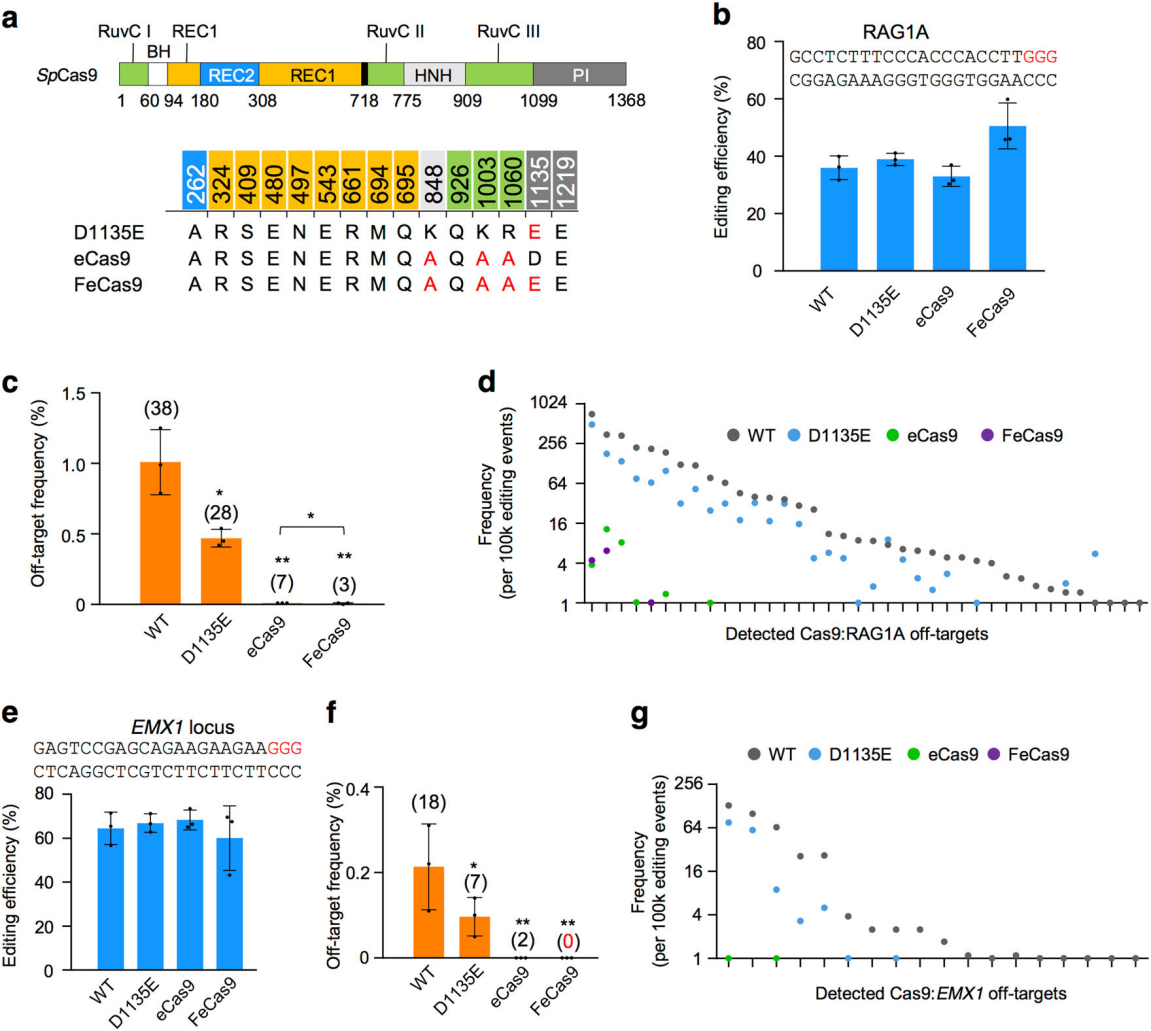
Editing efficiencies and specificity of Cas9 variants. **a** Schematic of Cas9 domains and corresponding point mutations for Cas9 variants. Red letters indicated mutated amino acids of indicated Cas9 variants. **b** Editing efficiencies detected by primer-extension-mediated sequencing for Cas9 variants targeting RAG1A in HEK293T cells. Target site sequence was listed above and the red letters indicated the protospacer adjacent motif sequence. Error bars, mean ± SD. Two-tailed *t*-test, *p < 0.05. **c** Frequencies of total translocation junctions in 1-kb regions around off-target hotspots for indicated variants targeting RAG1A in HEK293T cells. Total numbers of identified off-target hotspots for each Cas9 variant were showed above the bar. Error bars, mean ± SD. Two-tailed *t*-test, *p < 0.05; **p < 0.01. **d** Scatter plot of RAG1A off-target hotspots for indicated variants. *y* Axis showed frequency of each hotspot per 100,000 editing events (indels plus translocation). Note that the wild-type (WT) libraries presented in **b**–**d** were independently prepared from the ones in Figs. 1 and 2. Sequencing reads for the libraries in this figure was less (~60%) and the identified hotspots were only 38, but still more than linear amplification-mediated high-throughput genome-wide translocation sequencing. **e**–**g** Editing efficiencies and off-target hotspots for Cas9 variants targeting *EMX1* site in HEK293T cells, depicted as described in the legend to **b**–**d**

Various variants were developed to improve the editing specificity of Cas9 (Fig. 4a). In this context, eCas9^15^ showed high specificity due to mutations reducing nonspecific Cas9 and DNA contacts. We put eCas9 under the same promoter as WT Cas9 and directed it to target RAG1A locus in HEK293T cells. As anticipated, we detected only seven off-target sites for eCas9 with no obvious loss of editing efficiency (Fig. 4b–d, Supplementary Table S4). All the detected off-target hotspots for eCas9 ranked top on the list of RAG1A off-target hotspots (Fig. 4d).

Next, we sought to test whether reducing the PAM binding of eCas9 could destabilize Cas9-off-target contacts and thus to further improve its specificity. For this purpose, we generated a new variant FeCas9 by introducing D1135E^31^ mutation to eCas9 (Fig. 4a). Even though D1135E mutation retained most RAG1A off-target sites, FeCas9 showed higher specificity than eCas9 with only 3 detected off-target hotspots (Fig. 4c). In addition, FeCas9 exhibited comparable editing efficiency at RAG1A site as both WT Cas9 and eCas9 (Fig. 4b). We next tested the specificity of FeCas9 at other loci including one at *EMX1* and another one close to *c-MYC* gene. We detected 18 off-targets for *EMX1* site by WT Cas9 via PEM-seq, with a loss of 3 weak ones and a gain of 5 new sites compared to GUIDE-seq analysis in U2OS cells^10^ (Supplementary Fig. S3A and Supplementary Table S5). FeCas9 consistently showed very low off-target activity with no obvious change of editing efficiency at tested loci; with regards to editing specificity, FeCas9 was better than eCas9 at all tested sites (Fig. 4e–g, Supplementary Fig. S3b–3d and Supplementary Tables S5-S6).

### AcrIIA4 inhibitor suppresses Cas9 off-target activity less effectively

**Fig. 5:**
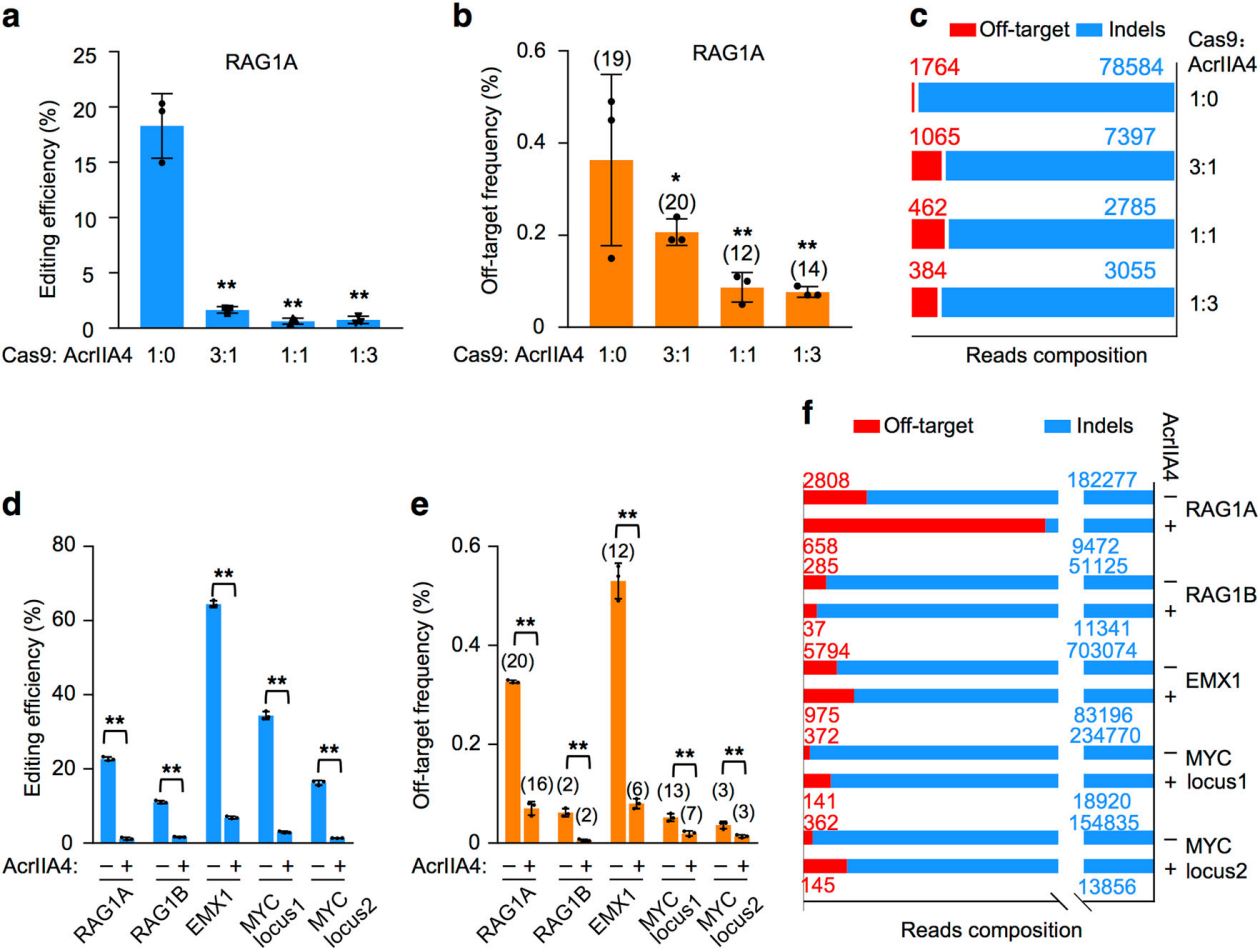
AcrIIA4 blocks Cas9 off-target activity less effectively. **a** Editing efficiencies detected by primer-extension-mediated sequencing for Cas9:RAG1A in HEK293T cells with indicated mass ratios of Cas9 over AcrIIA4. Error bars, mean ± SD. Two-tailed *t*-test, **p < 0.01. **b** Frequencies of translocation junctions in 1-kb regions around off-target hotspots for Cas9:RAG1A in HEK293T cells with indicated ratios of Cas9 over AcrIIA4. Total numbers of identified off-target hotspots for indicated samples were showed above the bars. Error bars, mean ± SD. Two-tailed *t*-test, *p < 0.05; **p < 0.01. **c** Composition of indels and off-target junctions for Cas9:RAG1A libraries with indicated ratios of Cas9 over AcrIIA4. Total junction numbers from pooled three libraries were indicated above the bars. Note that the total bar length of each library was normalized to the same though inhibitor-treated samples always contained less junctions than untreated ones. **d**–**f** Editing efficiencies and off-target hotspots in other Cas9-targeting loci with 1:1 Cas9 over AcrIIA4, depicted as described in the legend to **a**–**c**. Note that for 1:1 ratio described in **a**–**c**, the amount of plasmid DNA used for transfection for Cas9:RAG1A, AcrIIA4, and blank were 2, 2, and 4 μg, respectively; while for 1:1 in **d**–**f**, the amounts were 2 μg, 2 μg and 0, which led to a higher transfection efficiency of Cas9:RAG1A

We next used PEM-seq to demonstrate the ability of a widely used Cas9 inhibitor AcrIIA4 to block Cas9:RAG1A in HEK293T cells. We titrated the mass ratio of co-transfected Cas9:AcrIIA4 plasmids from 3:1 to 1:1 and finally 1:3. The editing efficiency of Cas9 decreased dramatically when AcrIIA4 was co-transfected. We found a 11-fold decrease of Cas9 editing efficiency at 3:1 and raising the ratio of AcrIIA4 to 1:1 and 1:3 further suppressed Cas9 activity (Fig. 5a). Correspondingly, AcrIIA4-treated Cas9 generated less off-target translocation junctions (Fig. 5b). However, the off-target activity decreased only 1.7–4.6 folds, not so dramatically as the loss of editing efficiency (Fig. 5c, Supplementary Fig. S4a, and Supplementary Table S7), suggesting that AcrIIA4 blocked Cas9 on-target activity more effectively than off-target activity. We also employed *Sa*Cas9 targeting *MYC* locus1 to unbiasedly capture both on- and off-target events of Cas9:RAG1A. The inhibitor had no impact to *Sa*Cas9 but caused a 22.8-fold decrease of Cas9 on-target activity while only 7-fold decrease for the off-target activity (Supplementary Fig. S4b–c and Supplementary Table S8), consistent with above finding.

We further tested five distinct loci with AcrIIA4 at 1:1 and detected significant suppression of Cas9 activity at all sites (Fig. 5d, e and Supplementary Table S9). Except for the RAG1B site containing too few off-target hotspots, the other four sites showed more robust suppression to on-target than off-target activity (Fig. 5f), in line with the finding for RAG1A site.

## Discussion

Here we developed PEM-seq to evaluate both editing efficiency and specificity of CRISPR/Cas9 in merely one Hiseq sequencing with 2–10 million reads. PEM-seq has two more advantages than currently used assays to assess CRISPR/Cas9 editing efficiency. First, primer extension and RMB in PEM-seq eliminate the amplification bias during PCR amplification used in other methods such as T7EI, RFLP, TIDE, and targeted sequencing. Second, PEM-seq detects small indels, large deletions, and genome-wide translocation, all of which are CRISPR/Cas9 editing events, while the above-mentioned three methods only detect small indels. PEM-seq also showed higher sensitivity to detect CRISPR/Cas9 off-target hotspots than LAM-HTGTS (Fig. 1), but improved HTGTS could also be very sensitive to detect off-target hotspots^32^. Compared to other methods designed only for off-target detecting, PEM-seq provides comprehensive information of CRISPR/Cas9 editing events, which definitely helps to choose appropriate target site or nucleases for genome editing. In this context, we showed that Cas9 nickases and AcrIIA4 inhibitor could be a better choice than Cas9 in consideration of only off-target activities, but it’s not true when taking both editing ability and off-target activities into account.

With regards to off-target damage, many factors can affect the final genome editing effect, such as target sequences, Cas9 variants, cell types, and Cas9 activation timing. In this context, FeCas9 could be a good candidate for genome editing (Fig. 4). In addition, control of activation timing could be an efficient way to rapidly revoke Cas9 activity, but co-transfection of Cas9 and AcrIIA4 inhibitor suppresses on-target activity severely than off-target damage, which may be due to that the NGG binding of Cas9 is not so crucial for cleavage at off-target sites but is key for efficient cleavage at on-target sites. In this context, delayed inhibitor delivery may be better since it retains high editing efficiency but nonetheless suppresses off-target activity^33^.

Besides off-target sites, other abnormal chromosomal structures derived from CRISPR/Cas9-induced DSBs also threaten genome stability. Here we showed that translocation could form when other DSBs including off-target DSBs and genome-wide low-level DSBs occur simultaneously with on-target DSBs, which could be tumor-driven when targeting oncogenes or tumor suppressors. In addition, inversions and deletions could also happen in the surrounding region of target sites^9^. Most of the deletions are very focal, but a detectable level of deletions can expand to 50 kb or an even larger region, which could lead to disruption of neighbor genes and thus cause unanticipated change to the edited cells. Since these abnormal structures are hallmarks of genome instability, PEM-seq can be easily adapted to study the function of various DNA repair factors in the DSB repair process, including the choice of repair pathway, the level of resection, formation of translocation, and so on.

### Limitation of PEM-seq

PEM-seq relies heavily on bait DSBs to capture genome-wide translocation. For the base editors^34,35^ that directly generate mutations in the genome, the current version of PEM-seq is not suitable for assessing their editing specificity. However, PEM-seq can be adapted to capture occasional DSB intermediates generated during base editing, similar to the DSBs formed in the process of class switch recombination induced by activation-induced cytidine deaminase^36^. Moreover, PEM-seq can identify off-target sites of CRISPR/Cas9 but cannot quantify the frequencies of identified off-target sites for two reasons: first, spatially proximity impacts formation of translocation and different off-targets show varied spatially proximity to the on-target sites^9,37^; second, PEM-seq detects indels only for the bait sites and thus cannot capture indels on the off-target sites if using on-target sites as bait, but extra PEM-seq analysis with off-target sites as bait can provide the indel information for off-target sites. Lastly, translocation is a rare event, therefore PEM-seq requires at least tens of thousands of cells to identify the off-target sites of given CRISPR/Cas9.

## Materials and methods

### PEM-seq procedure

#### Primer extension

All the biotinylated primers are placed within 200-bp from the cleavage site. For primer extension, repeated annealing and denaturation for biotin primer (by Sangon, Shanghai) and 20 μg sonicated genomic DNA (0.3–2 kb) was performed as following: 95 °C 3 min; 95 °C 2 min, *T*_a_ (annealing temperature) 3 min, 5 cycles; *T*_a_ 3 min. Then Bst polymerase 3.0 (NEB) was added to perform primer extension: 65 °C 10 min, 80 °C 5 min. Excessive biotinylated primers were depleted by 1.2× AxyPrep Mag PCR Clean-Up beads (Axygen, US). Purified products were heated to 95 °C for 5 min and then quickly chilled on ice for 5 min for DNA denaturation. Biotinylated PCR products were enriched by Dynabeads™ MyOne™ Streptavidin C1 (Thermo Fisher).

**Table:** 

Biotin primer	*T*_a_ (°C)
20–25 nt	50
25–30 nt	55
30–40 nt	58
40–50 nt	60

#### Bridge adapter (with RMB) ligation

PCR products on Streptavidin C1 beads were washed with 400 μl 1× B&W buffer twice (1 M NaCl, 5 mM Tris-HCl (pH 7.4), and 1 mM EDTA (pH 8.0)) followed by 400 μl dH_2_O washing. Then DNA-beads complex was resuspended with 42.4 μl dH_2_O. In the bridge adapter ligation step, ligation reaction was performed in 15% PEG8000 (Sigma) with T4 DNA ligase (Thermo Fisher Scientific) at room temperature overnight as following:

**Table:** 

DNA-beads	42.4 μl
10× T4 buffer	8 μl
Bridge adapter (50 μM)	1.6 μl
T4 DNA ligase (5 U/μl)	4 μl
50% (weight/volume) PEG8000	24 μl
Total	80 μl

#### PCR amplification for Illumina sequencing

Ligation products were washed with 400 μl 1× B&W buffer twice, 400 μl dH_2_O, and then resuspended with 80 μl dH_2_O. The beads-DNA complex underwent on-beads nested PCR (Taq, Transgen Biotech, China) with I5 and I7 sequence primers for 16 cycles. Then PCR products were recovered by size-selection beads (Axygen, US) followed by PCR (Fastpfu, Transgen Biotech, China) tagged with Illumina P5 and P7 sequence. All the PEM-seq libraries were subjected to sequencing by 2 × 150 bp Hiseq.

### Plasmid construction

All the gRNA sequences were listed (Supplementary Table S10). Cas9 targeting gRNAs used the pX330 backbone (Addgene 42230) and the Cas9 nickase using pX335 (Addgene 42335). Cas9 variants, *Sa*Cas9 were inserted into pX330 backbone as follows: Cas9 variants cDNAs (D1135E, eCas9(1.1), and FeCas9) were generated by mutation-overlap PCR and then inserted into pX330 plasmid through *Age*I/*Eco*RI. *Sa*Cas9 cDNA was purified with *Age*I/*Eco*RI from pX601 (Addgene 61591) and then directly ligated to *Age*I/*Eco*RI-digested pX330 plasmid, and the U6 promoter-*Sa*Cas9 gRNA scaffold DNA from pX601 was inserted into pX330-*Sa*Cas9 between *Afl*III and *Xba*I. AcrIIA4 plasmid PJH376 was obtained from Addgene (Addgene 86842).

### Cell line and cell transfection

293T, U2OS, and HCT116 cells were cultured in Dulbecco’s modified Eagle’s medium (Corning) with glutamine (Corning), 10% fetal bovine serum (FBS), and penicillin/streptomycin (Corning) at 37 °C with 5% CO_2_. K562 cells were cultured in RPMI 1640 (Corning) with glutamine, 15% FBS, and penicillin/streptomycin (Corning) at 37 °C with 5% CO_2_. Libraries for HEK293T cells were prepared by Ca-PO_4_ co-transfection with 7.2 μg nuclease plasmid and 1.8 μg pMAX-GFP in 6-cm dishes. Libraries for U2OS were co-transfected with 20 μg Cas9 plasmid and 5 μg green fluorescent protein (GFP) plasmid by PEI (Sigma) in 10 cm dishes. HCT116 were transfected with 20 μg Cas9 plasmid and 5 μg GFP plasmid by Lipofectamine 2000 (Invitrogen) in 10 cm dishes. Twenty micrograms of pX330 and 5 μg GFP were co-introduced into K562 cells by nucleofector 4D with FF120 program in SF buffer (Lonza). Cas9 inhibitor AcrIIA4 titration libraries were prepared from cells co-transfected with 2 μg Cas9:RAG1A plasmid, 6 μg AcrIIA4 plus blank plasmids in indicated ratios, and 1 μg GFP plasmid by Ca-PO_4_ in six-well dishes. For *Sa*Cas9:*MYC1* libraries, 2 μg *Sa*Cas9:*MYC* locus1 plasmid, 2 μg Cas9:RAG1A plasmid, 2 μg AcrIIA4 or blank plasmid, and 1 μg GFP plasmid were co-transfected into cells cultured in six-well dishes. For other 1:1 libraries, HEK293T cells were co-transfected with 2 μg pX330 plasmid, 2 μg AcrIIA4 or blank plasmid, and 1 μg GFP plasmid. All the libraries were analyzed for GFP co-transfection efficiency by fluorescence-activated cell sorting 48 h after plasmid delivery.

### “SuperQ” pipeline for PEM-seq analysis

Hiseq reads were processed as following. For initial reads preprocessing, both Illumina adapter sequences and ending low-quality sequences (QC < 30) were trimmed by cutadapt (http://cutadapt.readthedocs.io/en/stable/); remaining reads shorter than 25 bp were discarded. Then reads were de-multiplexed using fastq-multx (https://github.com/brwnj/fastq-multx) to distinguish index. For reads alignment and clustering, we adapted corresponding pipeline used in LAM-HTGTS^9^ to perform reads mapping and translocation break point detection. Note that we used the hg38 genome as reference. Uniquely mapped reads were filtered program as LAM-HTGTS did but all the duplicates were kept for following precession. A molecular barcode clustering algorithm^38^ was adapted to remove PCR duplicates at an editing distance of 2. Break-site and neighbor ±5 bp region was analyzed for indels; reads containing large deletions resulting from resection and rejoining in the break-site ±250 kb region were also categorized as indels (*I*). Reads without any detected mutations around break point were identified as germline (*G*). Genome-wide translocation (*T*) was identified as before. Taking uncut control and transfection efficiency (TE) into account, the editing efficiency was calculated as bellow:

$${\mathrm{Editing}}\,{\mathrm{efficiency}} = \frac{{\frac{{I_{\mathrm{S}} + T_{\mathrm{S}}}}{{G_{\mathrm{S}} + I_{\mathrm{S}} + T_{\mathrm{S}}}} - \frac{{I_{\mathrm{C}} + T_{\mathrm{C}}}}{{G_{\mathrm{C}} + I_{\mathrm{C}} + T_{\mathrm{C}}}}}}{{{\mathrm{TE}}}}$$

where S represents the nuclease-treated library and C represents the control library.

### Off-target hotspot identification

We excluded reads proximal to break-site (±250 kb) and used MACS2 *callpeak* mode to identify translocation enriched region: --extsize 50 -q 0.05 --llocal 10000000. MACS2 results were further filtered to remove sites with no target site-similar sequence or <3 junctions. A hotspot with highly similar sequence as target site and/or definite PAM and occurring in at least two library replicates was considered as an off-target site^9^. Briefly, the off-target hotspots are recurrent in a focal region containing cryptic target sequences and have a balanced directional distribution with a center right at the presumable cutting site. Yet, the off-target-independent translocations are usually low frequent and have biased directional distribution with no neighbor target-similar sequences. Total number of junctions within ±500 bp of off-target presumable cutting site was counted to calculate the off-target intensity after normalization to uncut control library:

$${\mathrm{Hotspot}}\,{\mathrm{intensity}} = \frac{{{\mathrm{Hotspot}}\,{\mathrm{junctions}}}}{{(I_{\mathrm{S}} - I_{\mathrm{C}}) + (T_{\mathrm{N}} - T_{\mathrm{C}})}} \times 100,000$$

### In vitro digestion for DNA fragments by Cas9

Cas9 was purified as described previously^39^. gRNA and scaffold RNA were transcribed by T7 High Efficiency Transcription Kit (Transgen Biotech, China) in vitro. A unit of 100 nM Cas9 and 300 nM RNA were used for each reaction. DNA fragments were digested in the condition: 20 mM HEPES (pH 7.5), 5% glycerol, 100 mM KCl, 1 mM dithiothreitol, 10 mM MgCl_2_, and 0.5 mM EDTA at 37 °C for 20 h.

### RFLP cleavage assay

RAG1A locus was PCR amplified by Fastpfu (Transgen Biotech, China) using listed primers (Supplementary Table S11). Amplicons recovered by 1.2× AxyPrep Mag PCR Clean-Up beads (Axygen, US) were cleaved by S*ty*I (NEB) for an hour followed by agarose gel electrophoresis. Band intensity was quantified by Image J (version 1.51J8) and the indels was measured by the following formula:

$${\mathrm{Indels}} = \frac{{I_{\mathrm{C}}}}{{I_{\mathrm{C}} + I_{\mathrm{U}}}}$$

*I*_C_ is sum of the intensity of two cleaved bands, and *I*_U_ is the intensity of the uncleaved bands.

### T7EI cleavage assay

Fraction cleaved (FC) ratio was calculated using the method described before^9^. Final indels was measured by the following formula (assuming the annealing prior to T7EI cutting is completely random):

$${\mathrm{Indels}} = 1 - \sqrt {1 - {\mathrm{FC}}}$$

### Single-cell RFLP cleavage assay

Single clone of Cas9:RAG1A transfected cells was picked into 96-well dish. Genomic DNA was extracted after 7-day incubation. And RFLP cleavage assay was performed as described in RFLP section. Cleavage products were classified into three categories: fully digested (*I*_I_), partially digested (*I*_H_), and non-digested (*I*_G_). Since the HEK293T cell we used is triploid, we scored fully digested as 3 and non-digested as 0. With regards to partially digested, either one or two alleles can be edited by Cas9 with equal chance in theory, and thus we scored them as 1.5. Therefore, the indels percentage was measured by the following formula:

$${\mathrm{Indels}} = \frac{{3 \times {{I}}_{\mathrm{I}} + 1.5 \times {{I}}_{\mathrm{H}}}}{{3 \times ({{I}}_{\mathrm{I}} + {{I}}_{\mathrm{H}} + {{I}}_{\mathrm{G}})}}$$

### TIDE assay

General process was referred to the method described before^23^. Primers were designed for RAG1A on-target site. Genomic DNA was extracted from Cas9:RAG1A transfected cells and 50 ng genomic DNA was used as template with routine PCR program for 30 cycles. Gel-purified PCR products were prepared for Sanger sequencing. The result file (*.ab1*) was analyzed by the website tool provided at https://tide.deskgen.com/.

### Statistical analysis

Data were presented as mean ± SD and *p* < 0.05 was considered significant.

### Additional resources

The “SuperQ” pipeline was deposited at github site: https://github.com/liumz93/superQ; and the PEM-seq data were deposited into GEO (GSE116231).

## Supplementary information

Supplementary Information

## Supplementary information

**Supplementary Information** accompanies the paper at (10.1038/s41421-019-0088-8).
