# Supplementary material for: Optimizing genome editing strategy by primer-extension-mediated sequencing
Source: Cell Discov. 2019 Mar 26;5:18. doi: 10.1038/s41421-019-0088-8 (PMC6434046; doi:10.1038/s41421-019-0088-8)
Supplement: Supplementary file 1 — Supplementary Information [file 41421_2019_88_MOESM1_ESM.pdf]

Supplementary Fig. S1

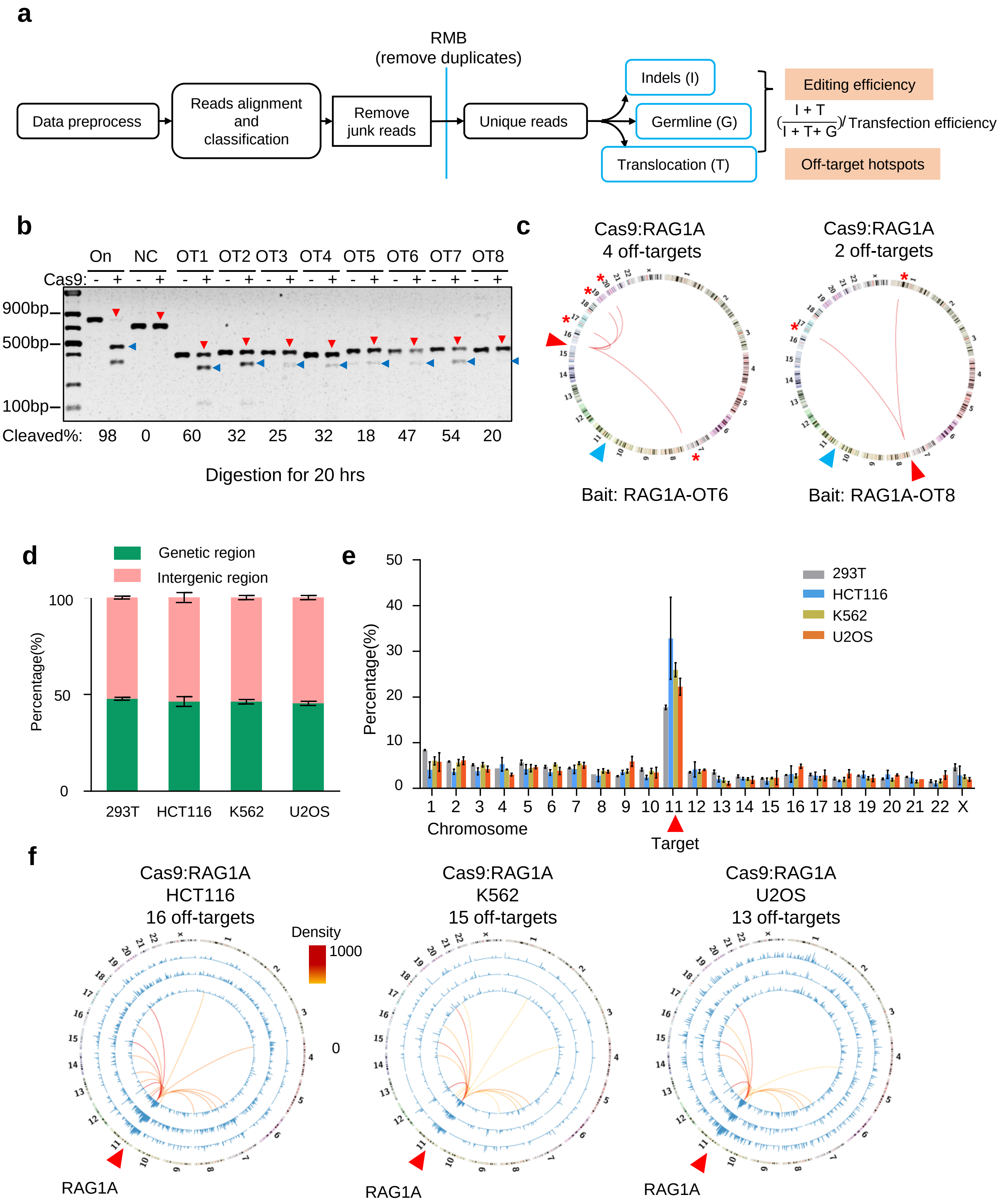

Supplementary Fig. S2

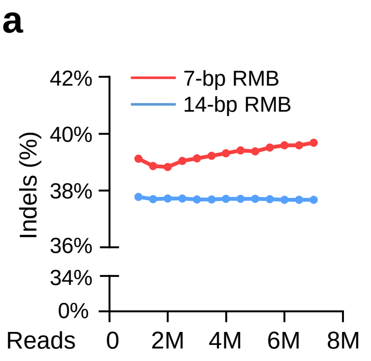

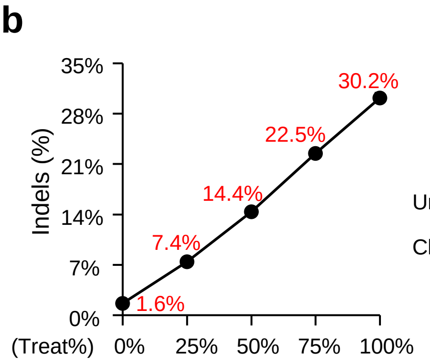

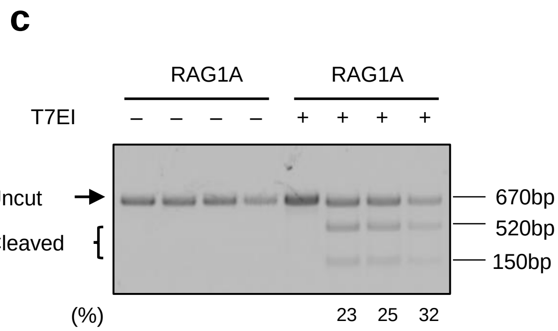

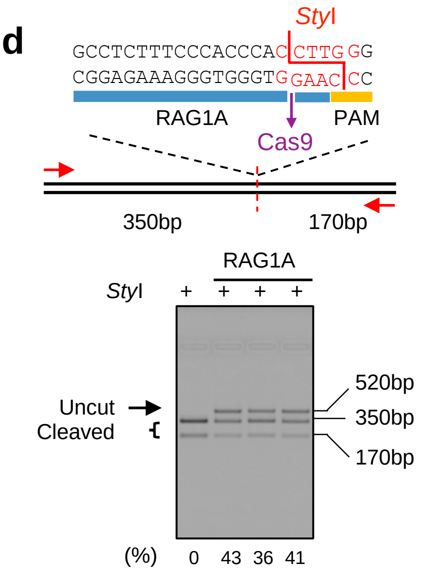

e

No.	F.D.	P.D.	N.D.	Indels%
1	34	87	46	46.4%
2	23	28	76	29.1%
3	16	27	64	27.6%

F.D.: Fully-digested;
P.D.: Partially-digested;
U.D.: Un-digested

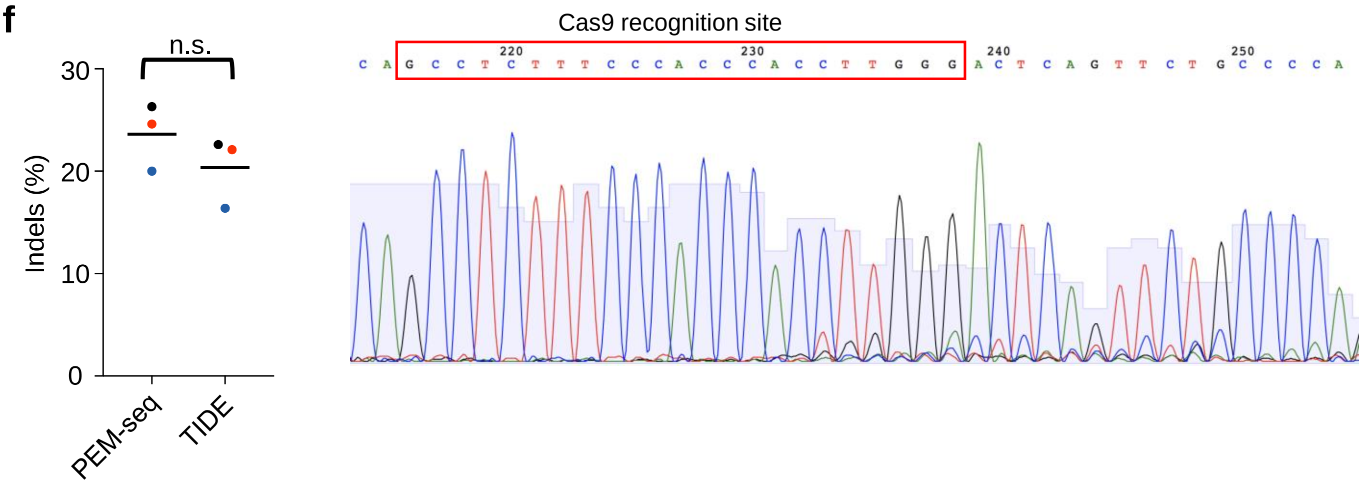

Supplementary Fig. S3

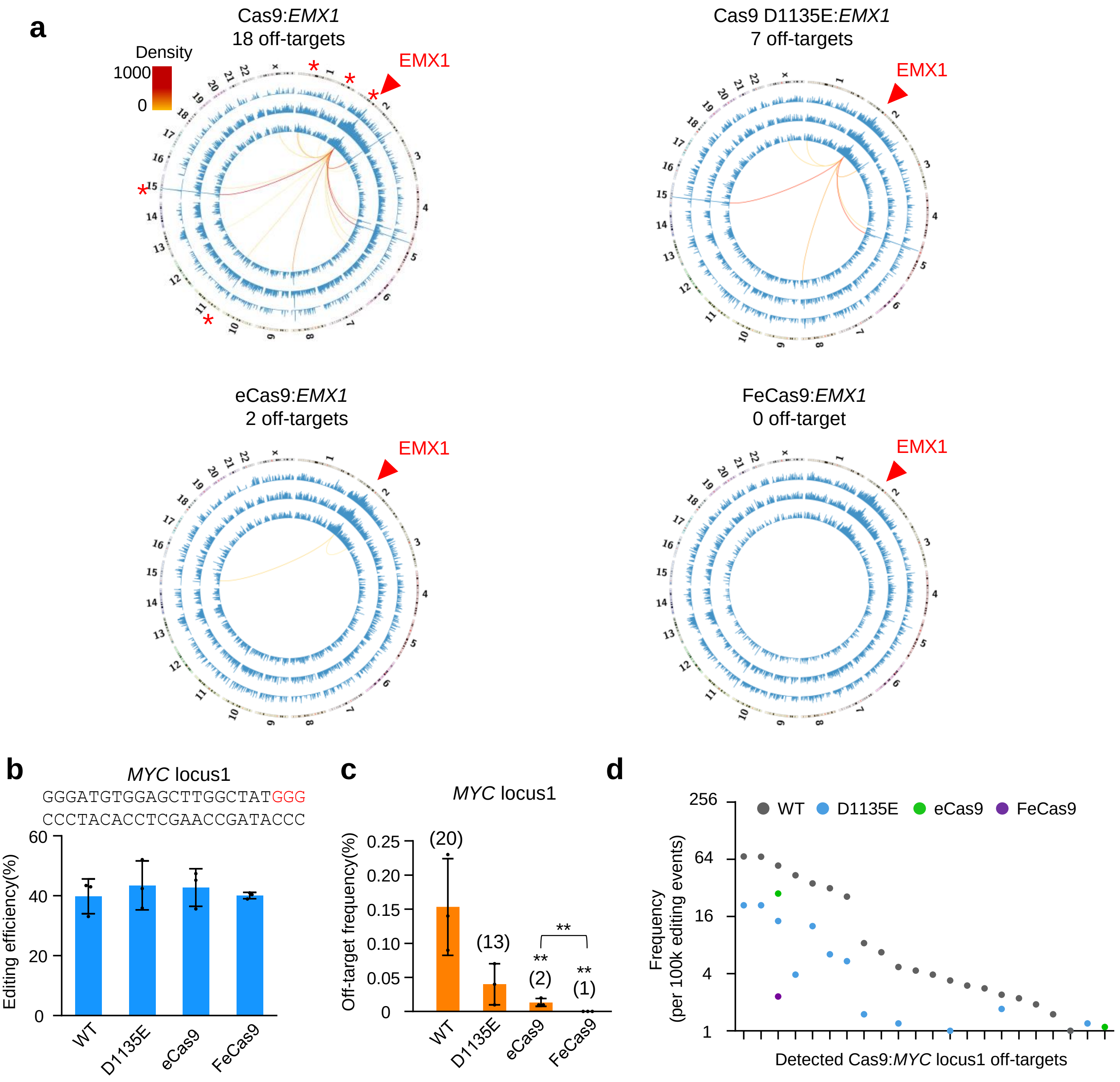

Supplementary Fig. S4

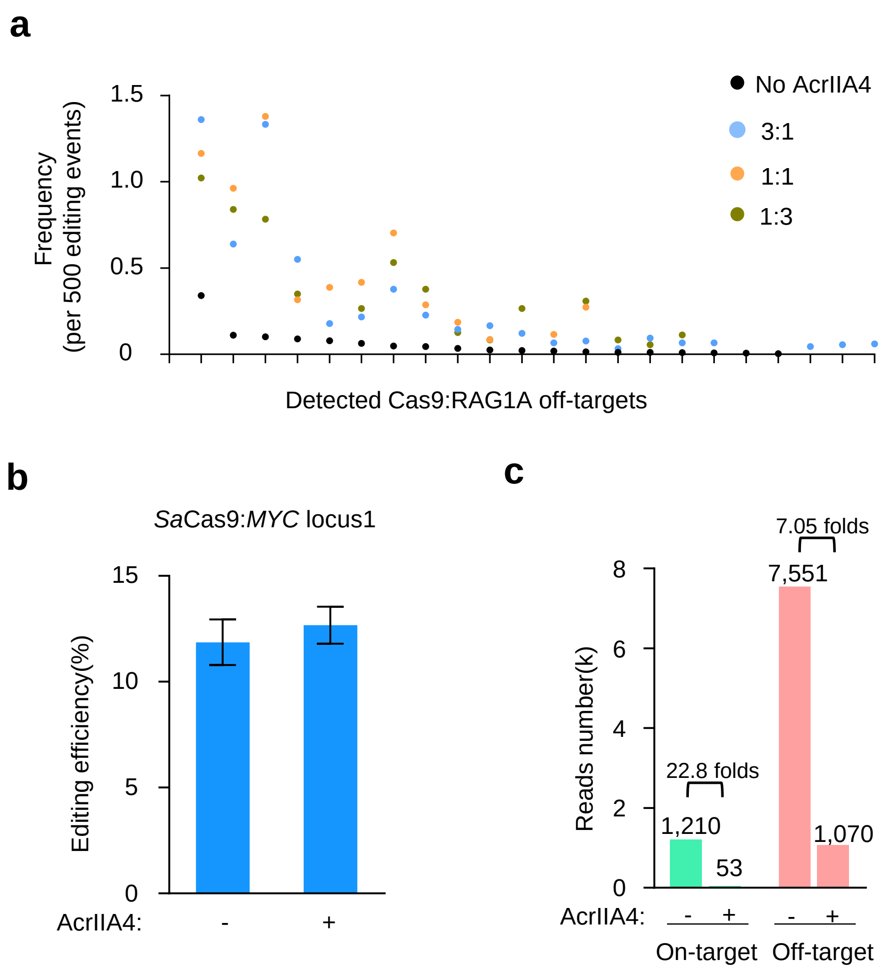

Figure legend

Fig. 1 PEM-seq Detects Off-target Hotspots of CRISPR/Cas9

(a) Overview of PEM-seq. To prepare PEM-seq libraries, primer extension generated a copy of template with biotinylated primer followed by bridge adapter ligation and DNA amplification. Gray bars represented bait region while orange ones for captured prey region; "N" indicated RMB in the bridge adapter. Arrows indicated positions and orientations of primers. See supplemental protocols for details.

(b) Circos plots of Cas9:RAG1A libraries. Three biological replicates were showed from outside to inside, and the displayed translocation junctions were 19,494, 16,005 and 18,078. Genome-wide translocation junctions binned into 5-Mb regions (blue lines) were plotted on a log scale. Chromosomes were showed with centromere to telomere in clockwise direction. Red arrow indicated the Cas9:RAG1A cleavage site. Colored lines connected the on-target site to the off-target hotspots.

(c) Zoomed-in view of Cas9:RAG1A translocation junctions (binned into 2Mb) on chromosome 7. Red arrows indicated identified off-target hotspots. Cen represented centromere and p/q indicated the chromosome arms. The Pearson correlation coefficient was 0.99 between replicates 1 and 2, 0.99 between replicates 1 and 3, 0.98 between replicates 2 and 3.

(d) Scatter plot of Cas9:RAG1A off-target hotspots in 293T, HCT116, K562 and U2OS cells. *y* axis showed frequency of each hotspot per 100,000 editing events (indels plus translocation). Red asterisks indicated the off-target hotspots detected by PEM-seq but not by LAM-HTGTS.

(e) Venn Diagram showing the overlap for RAG1A off-target hotspots among indicated cell lines. Legend is the same as showed in panel (d).

Fig. 2 Assessing Editing Efficiencies of CRISPR/Cas9 by PEM-seq

(a) Outcomes of Cas9-induced DSBs. Germline represents un-cut or perfect rejoining; indels is derived from errored rejoining; translocation involves a second DSB.

(b) Percentages of germline, indels and translocation showed by pie graph for Cas9:RAG1A. Mean ± SD.

(c) Frequency of indels for Cas9:RAG1A detected by PEM-seq, RFLP, T7EI assay and single cell RFLP. Averages were indicated by black lines. DNA from different batches were marked in different colors.
(d) Composition of Cas9:RAG1A translocation either joined with off-target DSBs or genome-wide low-level DSBs. Mean ± SD.
(e) Composition of indels of Cas9:RAG1A libraries within ±20 bp around cleavage-site. Note that part of deletions also involved insertion, indicated in gray. Mean ± SD.
(f) Frequency of junctions (binned into 50 bp) within ±5 kb (±20 bp excluded) around Cas9:RAG1A cleavage-site. Upper schematic showed different DNA repair products detected by PEM-seq, including inversion, deletion, and excision. Yellow box indicates the Cas9 target site. Blue and red arrows indicate the translocation orientations. Purple arrow indicates the position and orientation of primer used for PEM-seq. In the bottom panel, black dotted line indicates the Cas9 cleavage-site, while yellow dotted line indicates the primer locus. Junction numbers for each region were indicated.
(g) Frequency of junctions (binned into 1kb) for 5-50 kb downstream of Cas9:RAG1A cleavage-site. Black dotted lines indicate the boundaries of the 5-50 kb region. Purple arrow indicates the primer used for PEM-seq. Junction numbers for each region were indicated.

Fig. 3 Cas9 Nickase Shows Lower Off-target Activity with Loss of Target Editing Efficiency
Schematic of RAG1A, RAG1B, RAG1C and RAG1G target-site positions. Purple arrows indicated the biotinylated primers used for PEM-seq. Yellow boxes represented gRNA target sites and red bars indicated Cas9 cleavage site. In the lower panels, pie graph showed the compositions of germline, indels, and translocation for indicated Cas9 treatment in HEK293T cells. Mean ± SD.

Fig. 4 Editing Efficiencies and Specificity of Cas9 Variants
(a) Schematic of Cas9 domains and corresponding point mutations for Cas9 variants. Red letters indicated mutated amino acids of indicated Cas9 variants.
(b) Editing efficiencies detected by PEM-seq for Cas9 variants targeting RAG1A in HEK293T cells. Target site sequence was listed above and the red letters indicated the PAM

sequence. Error bars, mean ± SD. Two-tailed t-test, *, $p < 0.05$.

(c) Frequencies of total translocation junctions in 1-kb regions around off-target hotspots for indicated variants targeting RAG1A in HEK293T cells. Total numbers of identified off-target hotspots for each Cas9 variant were showed above the bar. Error bars, mean ± SD. Two-tailed t-test, *, $p < 0.05$; ** $p < 0.01$.

(d) Scatter plot of RAG1A off-target hotspots for indicated variants. *y* axis showed frequency of each hotspot per 100,000 editing events (indels plus translocation). Note that the WT libraries presented in (b-d) were independently prepared from the ones in Fig. 1 and 2. Sequencing reads for the libraries in this figure was less (~60%) and the identified hotspots were only 38, but still more than LAM-HTGTS.

(e-g) Editing efficiencies and off-target hotspots for Cas9 variants targeting *EMX1* site in HEK293T cells, depicted as described in the legend to panels b-d.

Fig. 5 AcrIIA4 Blocks Cas9 Off-target Activity Less Effectively

(a) Editing efficiencies detected by PEM-seq for Cas9:RAG1A in HEK293T cells with indicated mass ratios of Cas9 over AcrIIA4. Error bars, mean ± SD. Two-tailed t-test, **, $p < 0.01$.

(b) Frequencies of translocation junctions in 1-kb regions around off-target hotspots for Cas9:RAG1A in HEK293T cells with indicated ratios of Cas9 over AcrIIA4. Total numbers of identified off-target hotspots for indicated samples were showed above the bars. Error bars, mean ± SD. Two-tailed t-test, *, $p < 0.05$; ** $p < 0.01$.

(c) Composition of indels and off-target junctions for Cas9:RAG1A libraries with indicated ratios of Cas9 over AcrIIA4. Total junction numbers from pooled three libraries were indicated above the bars. Note that the total bar length of each library was normalized to the same though inhibitor-treated samples always contained less junctions than un-treated ones.

(d-f) Editing efficiencies and off-target hotspots in other Cas9-targeting loci with 1:1 Cas9 over AcrIIA4, depicted as described in the legend to panels (a-c). Note that for 1:1 ratio described in panels (a-c), the amount of plasmid DNA used for transfection for Cas9:RAG1A, AcrIIA4 and blank were 2 μg, 2 μg and 4 μg respectively; while for 1:1 in panels (d-f), the amounts were 2 μg, 2 μg and 0, which led to a higher transfection efficiency

of Cas9:RAG1A.

Supplementary Fig. S1 Genomic translocation features revealed by PEM-seq.

(a) Workflow of PEM-seq pipeline. Raw data was subjected to data preprocess, reads alignment and de-duplicates with RMB. Then unique reads were categorized into germline, indels and translocation according to the sequence features. See online methods for details.

(b) *In vitro* Cas9 digestion on RAG1A off-target hotspots. Indicated amplified fragments were incubated with purified Cas9 for 20 hrs. “On” was the RAG1A on-target site. “NC”, negative control with no Cas9:RAG1A target sites but can be targeted by Cas9:*MYC1*. Red triangles indicated the un-cleaved fragment, while blue triangles indicated the larger cleaved fragments. The detailed information of these off-targets were showed in Supplementary Table S2.

(c) Circos plots of Cas9:RAG1A libraries with RAG1A OT6 and OT8 loci as bait in 293T cells. Red triangles indicated the bait sites and red asterisks indicated the detected off-target, and blue triangles indicated the position of RAG1A on-target sites. The detailed information of these off-targets are showed in Supplementary Table S2.

(d) Frequencies of translocation junctions in genetic and intergenic regions for Cas9:RAG1A in indicated cell lines. Error bars, mean ± SD.

(e) Distribution profiles of off-target hotspots-excluded translocation junctions in each chromosome for Cas9:RAG1A in indicated cell lines. Red arrow indicated the target site-containing chromosome. Error bars, mean ± SD.

(f) Circos plots of Cas9:RAG1A libraries in HCT116, K562 and U2OS cell lines, depicted as described in the legend to Fig.1b.

Supplementary Fig. S2 Detecting indels generated by CRISPR/Cas9.

(a) Frequencies of indels detected by PEM-seq from titrated amounts of raw reads extracted from Cas9:RAG1A library with 14-bp or 7-bp RMB. The original read size for the Cas9:RAG1A library was 7.85 million. We randomly took 1 M to 7M (one sample per 0.5M) raw reads to go through the PEM-seq “SuperQ” pipeline individually. The 7-bp RMB was

truncated from 14-bp RMB for simulation analysis. If the RMB got saturated, the frequencies of indels would increase due to the miss-assignment of germline as showed by the red line.

(b) Frequencies of indels for libraries with indicated ratios of Cas9:RAG1A-treated and un-treated DNA. The frequency of indels consistently increased about 7% per 25% more Cas9-treated DNA.

(c) Frequencies of indels detected by T7EI for Cas9:RAG1A in HEK293T cells. Cas9:RAG1A cleavge site was PCR amplified and either mock cleaved (–) or T7EI cleaved (+). Length of the uncut and cleaved DNA, and frequencies of indels for each lane was indicated. See online methods for details.

(d) Frequencies of indels detected by RFLP for Cas9:RAG1A in HEK293T cells. Cas9:RAG1A targeting sequence was underlined by blue and PAM sequence by yellow. Purple arrow indicated the Cas9 cutting site. Red sequence indicated the endonuclease *Sty*I recognition site and red line showed the cutting site. Length of the uncut and *Sty*I-cleaved DNA, and frequencies of indels for each lane were indicated. See online methods for details.

(e) Table for single cell RFLP in HEK293T cells statistics. Three biological repeats were showed. See online methods for details.

(f) Frequencies of indels for Cas9:RAG1A detected by PEM-seq and TIDE. Note that DNA used here was different batch as that used in Fig. 2. Red box indicates the Cas9:RAG1A target site. DNA from the same batch were marked in the same color. n.s. not significant. See online methods for details.

Supplementary Fig. S3 Editing efficiency and off-target hotspots detected by PEM-seq for Cas9 variants.

(a) Circos plots of Cas9 variants for *EMX1* locus libraries, depicted as described in the legend to Fig. 1b.

(b-d) Editing efficiencies and off-target hotspots for Cas9 variants targeting *MYC* locus1 in HEK293T cells, depicted as described in the legend to panels Fig.4b-d.

Supplementary Fig. S4 AcrIIA4 suppresses Cas9 activity.

(a) Scatter plot of Cas9:RAG1A off-target hotspots in HEK293T cells with indicated ratios of Cas9 over AcrIIA4. *y* axis showed frequency of each hotspot per 500 editing events (indels + translocation). There low-frequency off-target hotspots detected in 3:1 libraries but not in no-inhibitor libraries were on the list of 53 total Cas9:RAG1A off-target sites. Only 19 off-target hotspots were detected in the no-inhibitor control libraries, because blank plasmid mock added into the transfection mixture reduced the delivery efficiency of Cas9:RAG1A plasmid.

(b) Editing efficiencies detected by PEM-seq for *Sa*Cas9:*MYC* locus1 with or without AcrIIA4 inhibitor. Error bars, mean ± SD.

(c) Translocation junctions captured for Cas9:RAG1A on- and off-target sites with or without AcrIIA4 inhibitor identified by *Sa*Cas9:*MYC* locus1 bait. Total numbers of Cas9:RAG1A on- and off-target junctions for each treatment were showed above the bar. Fold changes of junctions between different treatments were indicated.

Supplemental Tables

Supplementary Table S1: Information of PEM-seq libraries

RAG1A locus in different cell lines										
**Nuclease**	**Cell line**	**Locus**	**GFP (%)**	**DNA**	**Reads**	**Trans-location**	**Indels**	**Germline**	**Editing efficiency**	**Pearson correlation coefficient**
Cas9	293T	RAG1A	95%	20ug	7,851,886	19,494	290,742	460,327	39.7%	1.00
	293T	RAG1A	90%	20ug	6,632,310	16,005	236,954	459,240	36.5%	1.00
	293T	RAG1A	93%	20ug	6,939,348	18,078	237,726	406,670	39.0%	1.00
**Total**				**60ug**	**21,423,544**	**53,577**	**765,422**	**1,326,237**		
Cas9	HCT116	RAG1A	84%	20ug	2,594,049	1,002	34,315	453,894	6.1%	1.00
	HCT116	RAG1A	81%	20ug	3,093,171	1,161	34,702	466,884	6.2%	1.00
	HCT116	RAG1A	79%	20ug	2,687,388	647	30,707	465,548	5.3%	1.00
**Total**				**60ug**	**8,374,608**	**2,810**	**99,724**	**1,386,326**		
Cas9	K562	RAG1A	97%	20ug	5,714,664	5,564	177,063	998,723	13.8%	1.00
	K562	RAG1A	96%	20ug	5,171,097	4,977	129,098	869,775	11.8%	1.00
	K562	RAG1A	97%	20ug	5,088,244	4,586	139,803	899,627	12.2%	1.00
**Total**				**60ug**	**15,974,005**	**15,127**	**445,964**	**2,768,125**		
Cas9	U2OS	RAG1A	80%	20ug	4,069,162	2,496	60,684	746,677	7.2%	1.00
	U2OS	RAG1A	80%	20ug	3,837,414	2,122	56,258	693,266	7.2%	1.00
	U2OS	RAG1A	80%	20ug	3,652,282	2,019	52,057	657,544	7.0%	1.00
**Total**				**60ug**	**11,558,858**	**6,637**	**168,999**	**2,097,487**		
Other loci around RAG1A										
Cas9	293T	RAG1B	90%	20ug	3,080,750	1,654	90,793	435,451	18.8%	1.00
	293T	RAG1B	88%	20ug	3,212,193	1,295	79,374	376,688	19.4%	1.00
	293T	RAG1B	88%	20ug	2,126,589	927	48,035	200,496	21.7%	1.00
**Total**				**60ug**	**8,419,532**	**3,876**	**218,202**	**1,012,635**		
Cas9	293T	RAG1C	90%	20ug	1,201,887	988	73,467	200,535	28.2%	1.00
	293T	RAG1C	88%	20ug	1,997,275	1,552	89,084	271,415	26.6%	1.00
	293T	RAG1C	82%	20ug	3,017,360	1,784	137,037	401,451	29.3%	1.00
**Total**				**60ug**	**6,216,522**	**4,324**	**299,588**	**873,401**		
Cas9n	293T	RAG1A&G	92%	20ug	5,329,428	2,071	128,249	360,296	21.6%	1.00
	293T	RAG1A&G	86%	20ug	2,612,348	1,785	89,569	324,397	17.8%	1.00
	293T	RAG1A&G	83%	20ug	2,616,692	1,589	88,624	318,448	18.4%	1.00
**Total**				**60ug**	**10,558,468**	**5,445**	**306,442**	**1,003,141**		
RAG1A and DNA titration libraries										
4:0	293T	RAG1A	-	20ug	2,765,758	10,562	146,721	328,693	32.4%	-
3:1	293T	RAG1A	-	20ug	2,244,669	6,617	98,938	334,469	24.0%	-

2:2	293T	RAG1A	-	20ug	2,447,355	4,827	77,638	456,722	15.3%	-
1:3	293T	RAG1A	-	20ug	1,814,991	2,322	40,017	497,239	7.8%	-
0:4	293T	RAG1A	-	20ug	1,718,514	45	8,995	542,549	1.6%	-
Cas9:RAG1A variants										
Cas9	293T	RAG1A	87%	20ug	3,842,485	11,159	156,800	339,285	36.2%	1.00
	293T	RAG1A	89%	20ug	4,726,942	18,801	227,188	416,858	40.0%	1.00
	293T	RAG1A	80%	20ug	4,147,416	8,087	137,379	388,652	31.8%	1.00
**Total**				**60ug**	**12,716,843**	**38,047**	**521,367**	**1,144,795**		
D1135E	293T	RAG1A	98%	20ug	4,752,710	10,281	196,184	327,216	36.9%	1.00
	293T	RAG1A	96%	20ug	3,500,646	10,085	193,552	310,619	38.7%	1.00
	293T	RAG1A	96%	20ug	4,186,260	10,531	192,087	281,209	41.2%	1.00
**Total**				**60ug**	**12,439,616**	**30,897**	**581,823**	**919,044**		
eCas9	293T	RAG1A	92%	20ug	3,225,144	5,381	137,897	249,179	37.0%	1.00
	293T	RAG1A	90%	20ug	3,084,029	5,052	133,061	327,003	30.4%	1.00
	293T	RAG1A	88%	20ug	1,846,304	3,105	88,385	211,194	31.6%	1.00
**Total**				**60ug**	**8,155,477**	**13,538**	**359,343**	**787,376**		
FeCas9	293T	RAG1A	87%	20ug	2,227,781	13,222	59,680	162,041	46.0%	1.00
	293T	RAG1A	82%	20ug	2,476,621	17,906	86,764	188,102	59.8%	1.00
	293T	RAG1A	81%	20ug	2,181,993	11,605	47,357	139,839	45.9%	1.00
**Total**				**60ug**	**6,886,395**	**42,733**	**193,801**	**489,982**		
Cas9:EMX1 variants										
*Sp*Cas9	293T	EMX1	83%	20ug	2,259,843	3,561	182,614	202,850	57.9%	1.00
	293T	EMX1	96%	20ug	1,728,790	13,255	253,009	138,626	68.9%	1.00
	293T	EMX1	97%	20ug	1,617,812	6,148	196,836	80,635	73.6%	1.00
**Total**				**60ug**	**5,606,445**	**22,964**	**632,459**	**422,111**		
D1135E	293T	EMX1	86%	20ug	2,245,786	4,408	281,487	234,844	63.8%	1.00
	293T	EMX1	93%	20ug	1,442,261	5,278	219,216	128,526	68.2%	1.00
	293T	EMX1	98%	20ug	1,430,186	4,982	206,844	84,695	72.9%	1.00
**Total**				**60ug**	**5,118,233**	**14,668**	**707,547**	**448,065**		
eCas9	293T	EMX1	90%	20ug	2,825,769	3,978	252,905	168,454	67.4%	1.00
	293T	EMX1	94%	20ug	1,349,284	5,640	238,614	145,073	66.7%	1.00
	293T	EMX1	90%	20ug	1,577,042	4,374	189,306	93,930	75.2%	1.00
**Total**				**60ug**	**5,752,095**	**13,992**	**680,825**	**407,457**		
FeCas9	293T	EMX1	86%	20ug	2,015,130	4,328	213,106	257,933	53.4%	1.00
	293T	EMX1	96%	20ug	1,751,609	5,358	268,147	156,880	66.0%	1.00
	293T	EMX1	96%	20ug	1,530,437	2,797	170,044	146,198	56.7%	1.00
**Total**				**60ug**	**5,297,176**	**12,483**	**651,297**	**561,011**		
Cas9:*MYC*-locus1 variants										
Cas9	293T	*MYC*-locus1	97%	20ug	3,884,455	9,377	149,327	207,022	43.0%	1.00
	293T	*MYC*-locus1	87%	20ug	5,007,042	5,956	134,327	323,853	33.1%	1.00
	293T	*MYC*-	85%	20ug	3,040,133	8,301	178,424	297,689	43.4%	1.00

		locus1								
**Total**				**60ug**	**11,931,630**	**23,634**	**462,078**	**828,564**		
D1135E	293T	*MYC*-locus1	98%	20ug	3,759,229	11,409	215,672	204,814	52.1%	1.00
	293T	*MYC*-locus1	91%	20ug	1,410,295	4,519	113,828	175,876	42.4%	1.00
	293T	*MYC*-locus1	73%	20ug	3,409,507	6,840	188,745	509,110	35.9%	1.00
**Total**				**60ug**	**8,579,031**	**22,768**	**518,245**	**889,800**		
eCas9	293T	*MYC*-locus1	98%	20ug	4,222,772	9,585	172,112	195,976	47.4%	1.00
	293T	*MYC*-locus1	88%	20ug	3,047,053	6,952	141,436	302,895	35.6%	1.00
	293T	*MYC*-locus1	98%	20ug	4,155,923	7,005	141,635	174,922	45.2%	1.00
**Total**				**60ug**	**11,425,748**	**23,542**	**455,183**	**673,793**		
FeCas9	293T	*MYC*-locus1	98%	20ug	3,738,130	5,910	137,411	199,781	41.0%	1.00
	293T	*MYC*-locus1	89%	20ug	3,481,196	4,462	101,718	186,367	38.9%	1.00
	293T	*MYC*-locus1	98%	20ug	2,374,662	4,426	114,930	170,927	40.4%	1.00
**Total**				**60ug**	**9,593,988**	**14,798**	**354,059**	**557,075**		
AcrIIA4 inhibitor libraries										
1:0	293T	RAG1A	72%	20ug	1,513,624	2,713	37,828	207,508	20.3%	1.00
	293T	RAG1A	68%	20ug	1,259,867	2,357	33,008	199,679	19.6%	1.00
	293T	RAG1A	54%	20ug	1,081,871	825	17,400	167,655	15.0%	1.00
**Total**				**60ug**	**3,855,362**	**5,895**	**88,236**	**574,842**		
3:1	293T	RAG1A	76%	20ug	1,055,671	614	6,602	225,486	1.8%	0.98
	293T	RAG1A	76%	20ug	1,276,393	806	7,878	273,475	1.8%	0.98
	293T	RAG1A	54%	20ug	1,064,759	350	4,820	209,925	1.3%	0.98
**Total**				**60ug**	**3,396,823**	**1,770**	**19,300**	**708,886**		
1:1	293T	RAG1A	68%	20ug	1,253,450	377	5,776	278,637	0.7%	0.98
	293T	RAG1A	64%	20ug	1,038,947	293	5,237	239,284	0.9%	0.97
	293T	RAG1A	48%	20ug	1,214,018	100	4,703	252,257	0.4%	0.98
**Total**				**60ug**	**3,506,415**	**770**	**15,716**	**770,178**		
1:3	293T	RAG1A	68%	20ug	1,210,093	199	4,770	229,174	0.6%	0.97
	293T	RAG1A	69%	20ug	1,442,245	291	6,618	330,241	0.5%	0.97
	293T	RAG1A	62%	20ug	891,225	102	3,555	148,599	1.1%	0.97
**Total**				**60ug**	**3,543,563**	**592**	**14,943**	**708,014**		
1:0	293T	RAG1A	61%	20ug	2,958,042	4,174	69,064	423,828	22.3%	1.00
	293T	RAG1A	60%	20ug	3,545,677	3,962	72,852	430,571	23.3%	1.00
	293T	RAG1A	60%	20ug	2,538,328	3,129	56,638	344,469	22.5%	1.00

**Total**				**60ug**	**9,042,047**	**11,265**	**198,554**	**1,198,868**		
1:1	293T	RAG1A	56%	20ug	2,638,509	380	10,624	515,716	1.6%	0.97
	293T	RAG1A	55%	20ug	2,476,639	364	7,632	460,536	0.9%	0.96
	293T	RAG1A	58%	20ug	2,620,035	306	7,815	433,166	1.1%	0.96
**Total**				**60ug**	**7,735,183**	**1,050**	**26,071**	**1,409,418**		
1:0	293T	RAG1B	64%	20ug	1,336,396	380	16,561	192,913	10.8%	1.00
	293T	RAG1B	65%	20ug	1,668,598	369	19,288	217,289	10.9%	1.00
	293T	RAG1B	66%	20ug	1,809,127	430	23,597	249,305	11.5%	1.00
**Total**				**60ug**	**4,814,121**	**1,179**	**59,446**	**659,507**		
1:1	293T	RAG1B	68%	20ug	1,603,237	68	5,932	252,289	1.7%	1.00
	293T	RAG1B	75%	20ug	1,791,627	58	7,872	325,862	1.6%	0.99
	293T	RAG1B	70%	20ug	2,005,905	90	8,364	336,542	1.8%	0.99
**Total**				**60ug**	**5,400,769**	**216**	**22,168**	**914,693**		
1:0	293T	MYC1	57%	20ug	2,377,797	2,637	80,618	316,712	34.0%	1.00
	293T	MYC1	54%	20ug	2,468,967	2,581	80,095	334,090	33.6%	1.00
	293T	MYC1	58%	20ug	2,735,892	2,944	94,124	341,969	35.6%	1.00
**Total**				**60ug**	**7,582,656**	**8,162**	**254,837**	**992,771**		
1:1	293T	MYC1	52%	20ug	3,515,540	296	14,726	440,508	3.3%	1.00
	293T	MYC1	58%	20ug	2,374,608	137	8,351	251,701	2.9%	1.00
	293T	MYC1	55%	20ug	2,663,791	300	15,157	477,481	2.8%	1.00
**Total**				**60ug**	**8,553,939**	**733**	**38,234**	**1,169,690**		
1:0	293T	MYC3	80%	20ug	2,690,416	2,977	58,929	327,096	16.7%	1.00
	293T	MYC3	72%	20ug	2,871,109	2,450	62,019	380,840	16.6%	1.00
	293T	MYC3	77%	20ug	3,226,089	3,086	66,093	406,850	15.6%	1.00
**Total**				**60ug**	**8,787,614**	**8,513**	**187,041**	**1,114,786**		
1:1	293T	MYC3	68%	20ug	2,983,856	499	18,096	517,949	1.4%	0.99
	293T	MYC3	68%	20ug	2,805,560	278	15,322	444,385	1.3%	0.99
	293T	MYC3	69%	20ug	2,964,143	321	17,917	510,070	1.4%	1.00
**Total**				**60ug**	**8,753,559**	**1,098**	**51,335**	**1,472,404**		
1:0	293T	EMX1	84%	20ug	2,852,531	5,071	234,331	184757	63.8%	1.00
	293T	EMX1	84%	20ug	3,267,308	6,209	246,013	180120	65.5%	1.00
	293T	EMX1	84%	20ug	2,634,404	6,145	243,544	187049	64.1%	1.00
**Total**				**60ug**	**8,754,243**	**17,425**	**723,888**	**551,926**		
1:1	293T	EMX1	74%	20ug	3,384,228	651	30,153	432677	6.6%	1.00
	293T	EMX1	78%	20ug	3,537,851	1,121	39,552	504690	7.3%	1.00
	293T	EMX1	78%	20ug	3,508,240	861	38,750	520959	6.8%	1.00
**Total**				**60ug**	**10,430,319**	**2,633**	**108,455**	**1,458,326**		
**Total**				**60ug**	**8,551,291**	**11,523**	**518,688**	**1,176,615**		
Cas9:RAG1A	293T	RAG1A-OT32	-	10ug	2,849,117	277	7,790	464,411	-	-
	293T	RAG1A-OT32	-	10ug	1,851,189	225	5,299	357,882	-	-
Cas9:RA	293T	RAG1A-	-	10ug	4,722,803	80	21,076	338,448	-	-

G1A		OT29								
	293T	RAG1A-OT29	-	10ug	2,936,487	116	13,809	227,911	-	-

PEM-seq control libraries

	Cell line	Locus	DNA	Total reads	Translocation	Indels	Germline
Cas9 variants	293T	RAG1A	20ug	3,964,448	50	45,241	605,764
Cas9	293T	RAG1B	20ug	769,966	14	4,617	698,247
Cas9	293T	RAG1C	20ug	1,181,902	11	4,473	272,936
Cas9 inhibitor	293T	RAG1A	20ug	1,695,139	7	6,102	363,426
Cas9	HCT116	RAG1A	20ug	3,040,041	13	11,711	538,051
Cas9	K562	RAG1A	20ug	6,657,170	67	27,757	1,299,770
Cas9	U2OS	RAG1A	20ug	4,197,923	42	17,885	838,120
Cas9 variants	293T	EMX1	20ug	2,185,150	9	10,682	477,511
Cas9 variants	293T	MYC1	20ug	2,347,509	58	7,534	463,882
1:1 AcrIIA4	293T	RAG1A	20ug	1,239,225	7	3,273	279,986
1:1 AcrIIA4	293T	RAG1B	20ug	1,239,225	7	3,273	279,986
1:1 AcrIIA4	293T	EMX1	20ug	3,532,528	6	10704	654373
1:1 AcrIIA4	293T	MYC1	20ug	2,346,147	58	7,534	463,882
1:1 AcrIIA4	293T	MYC2	20ug	1,332,715	25	7,521	298,450
*Sa*Cas9 AcrIIA4	293T	MYC1	20ug	2,062,054	23	5,812	451,326
Cas9	293T	RAG1A-OT32	10ug	2,366,218	26	5,293	381,173
Cas9	293T	RAG1A-OT29	10ug	1,864,579	8	12,214	176,109

(The Pearson correlation coefficient is for replicates 1 and 2, 2 and 3, 1 and 3 from top to bottom for each group.)

Supplementary Table S2: Cas9:RAG1A off-targets in different cell lines, *in vitro* digestion for Cas9:RAG1A off-targets and identification for Cas9:RAG1A off-targets with baits from Cas9: RAG1A off-targets

Cas9:RAG1A off-targets in different cell lines						
**Chromosome**	**Off-targets**	**Sequence**	**293T**	**HCT116**	**K562**	**U2OS**
chr11	RAG1A	GCCTCTTTCCCACCCACCTT GGG	-	-	-	-
chr12	1	TCCTCCTCCCCACCCACCTT CAG	724.9	87.6	88.5	139.0
chr15	2	TCCTCTCTCCCACCCACCTC CGG	481.7	104.5	34.3	58.6
chr19	3	ACCCCTTCCCCACCTACCTT GGG	277.9	83.4	32.0	82.0
chr7	4	CCCTCCTTCCCACCCACTTT GGG	277.9	26.8	6.6	9.4
chr11	5	ACGTCTTCCCCACCCACCTG GGG	226.7	22.6	4.6	23.4
chr14	6	ACTTCCTTCCCACCCACCTT CAG	222.5	25.4	10.7	12.5
chr7	7	GCCTCTTCCACACCCACCCT GGG	164.5	17.0	10.2	12.5
chr1	8	TCCTCTTCTCCACCCACCTC TGG	127.4	15.5	-	-
chr17	9	GTCTCTTTCTCACCCACTTT GGG	76.3	26.8	4.1	6.3
chr7	10	CACCCTTTCCCATCCACCTT TGG	69.1	7.1	20.1	7.8
chr8	11	ACCTCCTTCCCGCCCACCTG GGG	68.1	2.8	2.3	7.0
chr11	12	TCCTCTTCTCCACCCACCAT AGG	63.3	15.5	3.1	2.3
chr20	13	GTCTCTTTCCCATCCACCTT TGA	56.4	-	3.1	-
chr13	14	GCCTCTTTACCACCTCACCT TGG	47.3	15.5	-	-
chr4	15	TTCTCTTCCCCACCCACCTT TGA	44.8	4.2	3.1	-
chr11	16	GCCCCTCCCCCACCCACCCT GGG	35.1	11.3	-	2.3
chrX	17	ACCACTTCCCCACCCACCTC AGG	18.5	-	-	-
chr17	18	GACTCTTTTCCACCCACCCT AGG	16.0	-	-	-
chr12	19	GCTTCTTCCCCACCCACACT TGG	12.1	11.3	-	-
chr3	20	ACCTCTTCCCTCCCCACCTT TGG	10.3	-	-	-
chr7	21	TGCCTCTCCCCACCCACCCT TGG	9.6	-	-	-
chr1	22	CACCCTTCCCCACCCACCCT GGG	9.3	-	1.02	-
chr17	23	ACCTCTTATCCACCCACCTT GGC	9.1	-	-	-
chr7	24	GCCCCTGCCCCACCCACCTG TGG	6.1	-	-	-
chr11	25	GCCCCTACCCCACCCACCTG GGT	5.8	-	1.27	-
chr5	26	TTTTCTTCCCCACCCACCTC GGG	5.8	-	-	3.12
chr22	27	TGCTCTTCCCCACCCACCCA AGG	4.9	-	-	-
chr16	28	GCCCCTTCCCCACCCACCCT GTG	3.3	-	-	-
chr14	29	GGCTCCTCCTCACCCACCTC TGG	2.5	-	-	-
chr11	30	TACCCTTCCCCATCCACCCT GGG	2.4	-	-	-
chr11	31	TCCCCTTCACCACCCACCTG AGG	2.4	-	-	-
chr3	32	GTCTCTTTCCCACCCACCTT GTT	2.4	-	-	-
chr11	33	TTATCTTTCCCAACCACCTT TGG	2.2	-	-	-
chr4	34	ACCTCTTTCCATCCCACCTT GGG	2.2	-	-	-
chr10	35	TCATCTTCCCCACCCACCGT GGG	2.1	-	-	-
chr15	36	CCCTCCTTCCCACCCACCTC CGA	1.9	-	-	-

chr11	37	ACCTCATCCCCACTCACCTG GGG	1.8	-	-	-
chr12	38	GCCTCTTTTCCACCCACCTA ATT	1.5	-	-	-
chr6	39	GTGTATTCCCCACCCACCTT GGG	1.5	-	-	-
chr8	40	ACCTCGTCCCCACCCACCCT GGA	1.5	-	-	-
chr3	41	GCCTCATCCACATCCACCTT CGG	1.3	-	-	-
chr11	42	GCCTCTCCGCCACCCACCAG AGG	1.2	-	-	-
chr7	43	GCCTCCTCCCCACCCACTCT GAG	1.2	-	-	-
chr16	44	CGCTCTGTCCCACCCACCTC CGG	1.1	-	-	-
chr11	45	ACATCAACCCCACCCACCGT AGG	0.9	-	-	-
chr12	46	GCCTCCTTCCCCTCCACCCC AGG	0.9	-	-	-
chr2	47	CTCCCCTTCCCACCCACCTC TGG	0.8	-	-	-
chr8	48	GCCTCATCCCCACCCACCCA AGG	0.8	-	-	-
chr11	49	GTCTCAAACCCACCCACCTT GGC	0.5	-	-	-
chr14	50	CCCCCTTTCCCACCCACCTT CTG	0.5	-	-	-
chr16	51	GCCTCAGTCCCACCCACCCC AGG	0.5	-	-	-
chr7	52	TCCTCTTCCTCTCCCACCTC CGG	0.5	-	-	-
chr16	53	TCCTCCTCTCCACCCACCTC TGG	0.2	-	-	-
In vitro digestion for Cas9:RAG1A off-targets						
**Chromosome**	**Off-targets**	**Sequence**				
chr12	OT1	TCCTCCTCCCCACCCACCTT CAG	Related to off-target #1 in Table S4			
chr14	OT2	ACTTCCTTCCCACCCACCTT CAG	Related to off-target #6 in Table S4			
chr8	OT3	ACCTCCTTCCCGCCCACCTG GGG	Related to off-target #12 in Table S4			
chr17	OT4	GACTCTTTTCCACCCACCCT AGG	Related to off-target #18 in Table S4			
chr10	OT5	AGCTCCTCCTCACCCACCTC GGG	Related to off-target #30 in Table S4			
chr16	OT6	GCCTCAGTCCCACCCACCCC AGG	Related to off-target #32 in Table S4			
chr17	OT7	CGCCTCTTACCATCCACCTT GGG	Related to off-target #38 in Table S4			
chr8	OT8	ACCTCGTCCCCACCCACCCT GGA	Related to off-target #29 in Table S4			
Cas9:RAG1A off-targets using baits from Cas9: RAG1A OT6 and OT8						
**Chromosome**	**Off-targets**	**Sequence**				
chr16	OT6	GCCTCAGTCCCACCCACCCC AGG	Related to off-target #32 in Table S4			
chr19	1	ACCCCTTCCCCACCTACCTT GGG	Related to off-target #2 in Table S4			
chr7	2	CCCTCCTTCCCACCCACTTT GGG	Related to off-target #4 in Table S4			
chr17	3	GTCTCTTTCTCACCCACTTT GGG	Related to off-target #9 in Table S4			
chr20	4	GTCTCTTTCCCATCCACCTT TGA	Related to off-target #11 in Table S4			
chr8	OT8	ACCTCGTCCCCACCCACCCT GGA	Related to off-target #29 in Table S4			
chr1	1	TCCTCTTCTCCACCCACCTC TGG	Related to off-target #7 in Table S4			
chr17	2	GTCTCTTTCTCACCCACTTT GGG	Related to off-target #9 in Table S4			

Supplementary Table S3: Off-targets for other Cas9:RAG1 sites

Chromosome	Off-targets	Sequence	Frequency
chr11	RAG1B	GACTTGTTTTCATTGTTCTC AGG	-
chr14	1	TCCTTGTTTTCATTGTTCTC TGG	581.9
chr4	2	CATTTGTTTTCATTGTTCTC TGG	69.2
chr11	RAG1A/G	GCCTCTTTCCCACCCACCTT GGG	-
chr12	1	TCCTCCTCCCCACCCACCTT CAG	4.1
chr11	2	ACGTCTTCCCCACCCACCTG GGG	3.2
chr7	3	CCCTCCTTCCCACCCACTTT GGG	1.8
chr19	4	ACCCCTTCCCCACCTACCTT GGG	1.8
chr13	5	GCCTCTTTACCACCTCACCT TGG	1.4

Supplementary Table S4: Off-targets for Cas9:RAG1A variants

Chromosome	Off-targets	Sequence	WT	D1135E	eCas9	FeCas9
chr11	RAG1A	GCCTCTTTCCCACCCACCTT GGG	-	-	-	-
chr12	1	TCCTCCTCCCCACCCACCTT CAG	713.8	497.3	3.8	4.4
chr19	2	ACCCCTTCCCCACCTACCTT GGG	348.1	179.8	13.0	6.2
chr11	3	ACGTCTTCCCCACCCACCTG GGG	336.5	137.7	8.2	-
chr7	4	CCCTCCTTCCCACCCACTTT GGG	221.3	75.5	1.0	-
chr15	5	TCCTCTCTCCCACCCACCTC CGG	212.6	65.8	1.0	0.9
chr14	6	ACTTCCTTCCCACCCACCTT CAG	188.0	99.0	1.4	-
chr1	7	TCCTCTTCTCCACCCACCTC TGG	123.0	31.8	-	-
chr7	8	GCCTCTTCCACACCCACCCT GGG	119.0	52.4	-	-
chr17	9	GTCTCTTTCTCACCCACTTT GGG	77.3	24.9	0.7	-
chr7	10	CACCCTTTCCCATCCACCTT TGG	65.4	31.8	-	-
chr20	11	GTCTCTTTCCCATCCACCTT TGA	45.5	17.8	-	-
chr8	12	ACCTCCTTCCCGCCCACCTG GGG	40.1	32.6		
chr13	13	GCCTCTTTACCACCTCACCT TGG	38.8	17.2	-	-
chr11	14	TCCTCTTCTCCACCCACCAT AGG	36.5	31.6	-	-
chr4	15	TTCTCTTCCCCACCCACCTT TGA	29.6	15.6	-	-
chr11	16	GCCCCTCCCCCACCCACCCT GGG	25.8	4.7	-	-
chr7	17	GCCTCTCCCCACCCACCCTT GGC	11.0	5.7	-	-
chr17	18	GACTCTTTTCCACCCACCCT AGG	10.3	4.7	-	-
chr3	19	ACCTCTTCCCTCCCCACCTT TGG	8.9	0.8	-	-
chr12	20	GCTTCTTCCCCACCCACACT TGG	8.7	1.8	-	-
chr1	21	CACCCTTCCCCACCCACCCT GGG	7.6	9.1	-	-
chr11	22	GCCCCTACCCCACCCACCTG GGT	6.5	4.5	-	-
chr16	23	GCCCCTTCCCCACCCACCCT GTG	6.1	2.4	-	-
chr7	24	GCCCCTGCCCCACCCACCTG TGG	5.8	1.6	-	-
chrX	25	ACCACTTCCCCACCCACCTC AGG	4.9	2.8	-	-
chr14	26	GGCTCCTCCTCACCCACCTC TGG	4.9	-	-	-
chr17	27	ACCTCTTATCCACCCACCTT GGC	4.3	0.8	-	-
chr22	28	GCCCCTTACCCATCCACCCT GGG	4.0	-	-	-
chr8	29	ACCTCGTCCCCACCCACCCT GGA	2.5	-	-	-
chr10	30	AGCTCCTCCTCACCCACCTC GGG	2.4	-	-	-
chr11	31	TACCCTTCCCCATCCACCCT GGG	1.8	-	-	-
chr16	32	GCCTCAGTCCCACCCACCCC AGG	1.6	-	-	-
chr22	33	TGCTCTTCCCCACCCACCCA AGG	1.5	2.0	-	-
chrX	34	CCTTCTTTCCCACCCACATG GGG	1.5	-	-	-
chr5	35	TTTTCTTCCCCACCCACCTC GGG	0.9	5.5	-	-
chr10	36	TCATCTTCCCCACCCACCGT GGG	0.7	-	-	-
chr11	37	TTATCTTTCCCAACCACCTT TGG	0.5	-	-	-
chr17	38	CGCCTCTTACCATCCACCTT GGG	0.5	-	-	-
chr4	39	GCCTCCCTCCCAACCAGGAT GAT	-	-	2.7	-

Supplementary Table S5: Off-targets for Cas9:EMX1 variants

Chromosome	Off-targets	Sequence	WT	D1135E	e*Sp*Cas9	Fe*Sp*Cas9
chr2	*EMX1*	GAGTCCGAGCAGAAGAAGAA GGG	-	-	-	-
chr15	1	GAGTCTAAGCAGAAGAAGAA GAG	129.3	75.1	0.8	-
chr5	2	GAGTTAGAGCAGAAGAAGAA AGG	100.0	59.3	-	-
chr2	3	GAGGCCGAGCAGAAGAAAGA CGG	64.9	8.9	0.5	-
chr5	4	AAGTCTGAGCACAAGAAGAA TGG	25.8	3.3	-	-
chr8	5	GAGTCCTAGCAGGAGAAGAA GAG	26.4	5.0	-	-
chr1	6	AAGTCCGAGGAGAGGAAGAA AGG	3.8	0.7	-	-
chr11	7	AAGCCCGAGCAAAGGAAGAA AGG	2.5	-	-	-
chrX	8	GAGTCCGGGAAGGAGAAGAA AGG	2.5	0.9	-	-
chr5	9	GAGCCGGAGCAGAAGAAGGA GGG	2.5	-	-	-
chr1	10	GAGCCTGAGCAGAAGGAGAA GGG	1.7	-	-	-
chr15	11	AAGTCCCGGCAGAGGAAGAA GGG	1.1	-	-	-
chr2	12	AAGGCAGAGCAGAGGAAGAG AGG	0.9	-	-	-
chr2	13	GAGTCTAAGCAGGAGAATAA AGG	1.1	-	-	-
chr6	14	ACGTCTGAGCAGAAGAAGAA TGG	0.8	-	-	-
chr15	15	GAGCCTGAGGAACAGGAGGA GGA	0.5	-	-	-
chr3	16	GAATCCAAGCAGGAGAAGAA GGA	0.6	-	-	-
chr1	17	GATTCCTACCAGAAGAAGAA TGG	0.5	-	-	-
chr13	18	GAGTAGGAGCAGGAGAAGAA GGA	0.5	-	-	-

Supplementary Table S6: Off-targets for Cas9:*MYC1* variants

Chromosome	Off-targets	Sequence	WT	D1135E	e*Sp*Cas9	Fe*Sp*Cas9
chr8	*MYC*-1	GGGATGTGGAGCTTGGCTAT GGG	-	-	-	-
chr8	1	AGGAAGTGGAGCTTGGCCTT GGG	68.2	21.0	-	-
chr8	2	AGGAAGTGGAGCTTGGCCTT GGG	67.8	21.0	-	-
chr9	3	GGGGTGTGGAGCTTGACTAT GAG	54.7	14.3	27.8	2.3
chr4	4	AGGATGAAGAGATTGGCTAT GGG	43.3	3.9	-	-
chr3	5	GGGAAGTGGAACCTGGCTCT GGG	35.7	12.7	-	-
chr12	6	TGGATGTGCAGCCTGGCTAT TGG	31.6	6.4	-	-
chr12	7	GGTATGCAGAGCTTGGCTTT CGG	25.8	5.4	-	-
chr18	8	GAGGTGTGGAGCTTGGTTAC TGG	8.4	1.5	-	-
chr1	9	GGGTTGCAGAGCTTGGCTAC GGG	6.7	-	-	-
chr2	10	GGGGTGTGGAGCATGGCTTT GGG	4.7	1.2	-	-
chr14	11	GGTGTGTGGAGCATGGCTAT TGG	4.3	-	-	-
chr15	12	TGAATGTGGAACTTGGCTAT TAG	3.9	-	-	-
chr20	13	AATATGTGGAGCTTGGCTAT AGC	3.4	0.8	-	-
chr10	14	GGGAAGAGGAGCCTGGCTTT GGG	3.0	-	-	-
chr2	15	GGGACTTGGAGCTTGGCTTT AGG	2.8	-	-	-
chr12	16	AGGGTCTGGAGCTTGGCTAA GGG	2.4	1.7	-	-
chr17	17	AGGATGTGGAGCTTGGATAT CAG	2.2	-	-	-
chr1	18	AAGATGTGGAACTTGGTTAC GGG	1.9	-	-	-
chr8	19	GAGATAAGGCCCTTGGCTAT GGG	1.5	-	-	-
chr2	20	GG-ATGAGGAG[G]CTTGGCTA- GGG	0.9	0.6	-	-
chr8	21	AGGAGATGGAGCCTGGCTTA GGG	-	1.2	-	-
chr20	22	GAGATGTGGAGCCTGACTAT GGA	-	-	1.1	-
chr1	23	AAGACATGGAGCCTGGCTAT GGT	-	-	-	-
chr20	24	AAGACTAGGATCTTGGATAA GGG	-	-	-	-

Supplementary Table S7: Off-targets for Cas9:RAG1A locus with AcrIIA4 inhibitor

Chromosome	Off-targets	Sequence	1:0	3:1	1:1	1:3
chr11	RAG1A	GCCTCTTTCCCACCCACCTT GGG	-	-	-	-
chr12	1	TCCTCCTCCCCACCCACCTT CAG	3.4	13.6	11.6	10.2
chr14	2	ACTTCCTTCCCACCCACCTT CAG	1.1	6.4	9.6	8.4
chr11	3	ACGTCTTCCCCACCCACCTG GGG	1.0	13.3	13.8	7.8
chr7	4	CCCTCCTTCCCACCCACTTT GGG	0.9	5.5	3.2	3.5
chr19	5	CCCCTTCCCC-ACCTACCTT GGG	0.8	1.8	3.9	-
chr15	6	TCCTCTCTCCCACCCACCTC CGG	0.6	2.2	4.2	2.7
chr7	7	GCCTCTTCCACACCCACCCT GGG	0.5	3.8	7.0	5.3
chr1	8	TCCTCTTCTCCACCCACCTC TGG	0.5	2.3	2.9	3.8
chr17	9	GTCTCTTTCTCACCCACTTT GGG	0.4	1.4	1.9	1.3
chr7	10	CACCCTTTCCCATCCACCTT TGG	0.3	1.7	0.9	0.8
chr13	11	GCCTCTTTACCACC[T]CACCTT GGG	0.2	1.2	-	2.7
chr11	12	TCCTCTTCTCCACCCACCAT AGG	0.2	0.7	1.2	-
chr8	13	ACCTCCTTCCCGCCCACCTG GGG	0.1	0.8	2.7	3.1
chr12	14	GCTTCTTCCCCACCCACACT TGG	0.1	0.3	-	0.8
chr20	15	GTCTCTTTCCCATCCACCTT TGA	0.1	0.9	-	0.6
chr11	16	CGCCCCTCCCC[C]ACCCACCCT GGG	0.1	0.7	-	1.1
chr4	17	TTCTCTTCCCCACCCACCTT TGA	0.1	0.7	-	-
chr11	18	GCCCCTACCCCACCCACCTG GGT	0.1	-	-	-
chr17	19	GACTCTTTTCCACCCACCCT AGG	0.1	-	-	-
chr11	20	CCTCGTGATCCACCCACCTC GGG	-	0.4	-	-
chr1	21	CACCCTTCCCCACCCACCCT GGG	-	0.6	-	-
chr17	22	ACCTCTTATCCACCCACCTT GGC	-	0.6	-	-

Supplementary Table S8: Off-targets for Cas9:RAG1A locus with AcrIIA4 inhibitor, and the bait DSBs were generated by *Sa*Cas9:*MYC1*

Chromosome	Off-targets	Sequence	1:0	1:1
chr11	RAG1A	GCCTCTTTCCCACCCACCTT GGG	616.0	33.8
chr8	1	TCCTCCTCCCCACCCACCTT CAG	1367.9	295.0
chr12	2	ACTTCCTTCCCACCCACCTT CAG	468.8	55.5
chr7	3	ACGTCTTCCCCACCCACCTG GGG	269.3	38.3
chr14	4	CCCTCCTTCCCACCCACTTT GGG	255.0	33.8
chr15	5	CCCCTTCCCC-ACCTACCTT GGG	220.9	33.8
chr7	6	TCCTCTCTCCCACCCACCTC CGG	181.7	19.8
chr1	7	GCCTCTTCCACACCCACCCT GGG	179.2	29.4
chr19	8	TCCTCTTCTCCACCCACCTC TGG	172.6	24.9
chr17	9	GTCTCTTTCTCACCCACTTT GGG	123.2	56.8
chr7	10	CACCCTTTCCCATCCACCTT TGG	103.8	20.4
chr13	11	GCCTCTTTACCACC[T]CACCTT GGG	75.9	7.7
chr20	12	TCCTCTTCTCCACCCACCAT AGG	66.7	3.8
chr11	13	ACCTCCTTCCCGCCCACCTG GGG	46.8	10.9
chr4	14	GCTTCTTCCCCACCCACACT TGG	36.1	8.9
chr12	15	GTCTCTTTCCCATCCACCTT TGA	34.6	7.0
chr7	16	CGCCCCTCCCC[C]ACCCACCCT GGG	31.1	17.9
chr11	17	TTCTCTTCCCCACCCACCTT TGA	28.5	7.7
chr17	18	GCCCCTACCCCACCCACCTG GGT	23.9	3.2
chr11	19	GACTCTTTTCCACCCACCCT AGG	21.4	3.8
chr17	20	CCTCGTGATCCACCCACCTC GGG	19.9	-
chr1	21	CACCCTTCCCCACCCACCCT GGG	14.3	-
chr22	22	ACCTCTTATCCACCCACCTT GGC	12.2	-
chrX	23	ACCACTTCCCCACCCACCTC AGG	12.2	4.5
chr2	24	CTCCCCTTCCCACCCACCTC TGG	9.7	-
chr8	25	GCCTCATCCCCACCCACCCA AGG	8.1	-
chr7	26	GCCCCTGCCCCACCCACCTG TGG	7.6	-
chr7	27	ACCTCCTAACCCCCCACCTT GGG	6.6	-
chr8	28	ACCTCGTCCCCACCCACCCT GGA	6.6	-
chr16	29	TCCTCCTCTCCACCCACCTC TGG	6.1	-
chr22	30	GCCCCTTACCCATCCACCCT GGG	6.1	-
chr10	31	TCATCTTCCCCACCCACCGT GGG	5.6	-
chr16	32	GCCTCAGTCCCACCCACCCC AGG	5.1	-
chr3	33	ACCTCTTCCCTCCCCACCTT TGG	4.1	-
chr1	34	TCTTCTCCCCCACCCACCCT GGG	3.6	-
chr6	35	ACCCCCTTCCCACCCACCCT GAG	3.6	-
chr8	36	TCCTCCCTCCCACCCACCTG AGG	3.6	-
chr8	37	GCCTCTTTCCTGCCCACCTG TGG	1.5	-

Supplementary Table S9: Off-targets for Cas9 with 1:1 AcrIIA4

Chromosome	Off-targets	Sequence	1:0	1:1
chr11	RAG1A	GCCTCTTTCCCACCCACCTT GGG		
chr12	1	TCCTCCTCCCCACCCACCTT CAG	1.9	5.8
chr11	2	ACGTCTTCCCCACCCACCTG GGG	0.9	4.4
chr19	3	ACCCCTTCCCCACCTACCTT GGG	0.8	1.4
chr7	4	CCCTCCTTCCCACCCACTTT GGG	0.7	2.0
chr14	5	ACTTCCTTCCCACCCACCTT CAG	0.6	3.6
chr15	6	TCCTCTCTCCCACCCACCTC CGG	0.5	1.8
chr17	7	GTCTCTTTCTCACCCACTTT GGG	0.2	1.4
chr7	8	GCCTCTTCCACACCCACCCT GGG	0.2	0.9
chr1	9	TCCTCTTCTCCACCCACCTC TGG	0.2	1.1
chr11	10	TCCTCTTCTCCACCCACCAT AGG	0.2	-
chr7	11	CACCCTTTCCCATCCACCTT TGG	0.2	1.2
chr13	12	GCCTCTTTACCACC[T]CACCTT GGG	0.1	1.3
chr20	13	GTCTCTTTCCCATCCACCTT TGA	0.1	0.5
chr8	14	ACCTCCTTCCCGCCCACCTG GGG	0.1	0.9
chr11	15	GCCCCTCCCCCACCCACCCT GGG	0.1	0.6
chr4	16	TTCTCTTCCCCACCCACCTT TGA	0.1	0.4
chr17	17	ACCTCTTATCCACCCACCTT GGC	0.1	0.1
chrX	18	ACCACTTCCCCACCCACCTC AGG	0.1	-
chr11	19	GCCCCTACCCCACCCACCTG GGT	0.1	-
chr17	20	GACTCTTTTCCACCCACCCT AGG	0.1	-
**Chromosome**	**Locus**	**Sequence**	**1:0**	**1:1**
chr11	RAG1B	GACTTGTTTTCATTGTTCTC AGG		-
chr14	1	TCCTTGTTTTCATTGTTCTC TGG	2.5	1.1
chr4	2	CATTTGTTTTCATTGTTCTC TGG	0.2	0.4
**Chromosome**	**Off-targets**	**Sequence**	**1:0**	**1:1**
chr8	*MYC*-locus1	GGGATGTGGAGCTTGGCTAT GGG	-	-
chr9	1	GGGGTGTGGAGCTTGACTAT GAG	0.2	0.4
chr8	2	AGGAAGTGGAGCTTGGCCTT GGG	0.1	0.6
chr8	3	AGGAAGTGGAGCTTGGCCTT GGG	0.1	0.6
chr4	4	AGGATGAAGAGATTGGCTAT GGG	0.1	0.2
chr12	5	GGTATGCAGAGCTTGGCTTT CGG	0.1	0.4
chr12	6	TGGATGTGCAGCCTGGCTAT TGG	0.1	0.4
chr3	7	GGGAAGTGGAACCTGGCTCT GGG	0.1	0.6
chr18	8	GAGGTGTGGAGCTTGGTTAC TGG	0.1	-
chr20	9	AAGACTAGGATCTTGGATAA GGG	0.1	-
chr2	10	GGGGTGTGGAGCATGGCTTT GGG	0.1	-
chr14	11	GGTGTGTGGAGCATGGCTAT TGG	0.1	-
chr15	12	TGAATGTGGAACTTGGCTAT TAG	0.1	-
chr17	13	AGGATGTGGAGCTTGGATAT CAG	0.1	-

Chromosome	Locus	Sequence	1:0	1:1
chr8	*MYC*-locus2	GTACATGCAGTTCTGCATCT TGG	-	-
chr6	1	ATAAATACAGTTCTGCATCT GGG	1.0	3.6
chr2	2	GTAAATGCAGTTCTGCATTT AGG	0.1	0.9
chr5	3	GCACATGCAGCCTTGCATCT GGG	0.1	0.4

Supplementary Table S10: gRNA sequences for PEM-seq

Locus	Sequence
RAG1A	GCCTCTTTCCCACCCACCTT (GGG)
RAG1B	GACTTGTTTTCATTGTTCTC (AGG)
RAG1C	GCACCTAACATGATATATTA (AGG)
RAG1G	GAAAGAGGCTGCCATGCTGGCTG (AGG)
EMX1	GAGTCCGAGCAGAAGAAGAA (GGG)
MYC-locus1	(TTTA) GGGATGTGGAGCTTGGCTAT (GGGAAT)
MYC-locus2	(TTTA) GTACATGCAGTTCTGCATCT (TGGGGT)

Supplementary Table S11: Oligos used for PEM-seq

Name	Sequence5'→3'	Purpose
**Bridge adapters oligos (PEM-seq)**		
BA-up	/5phos/CCA CGC GTG CTC TAC ANN NNT NNN ANN NTN NNN AGA TCG GAA GAG CAC ACG TCT GAA CTC CAG T-NH2(C7)	link ssDNA with RMB
BA-lower	TGT AGA GCA CGC GTG GNN NNN N-NH2(C7)	common bridge oligo
**Oligos for tagging PCR**		
P5-I5	AAT GAT ACG GCG ACC ACC GAG ATC TAC ACA CAC TCT TTC CCT ACA CGA CGC	Hiseq tail
P7	CAA GCA GAA GAC GGC ATA CGA GAT	Hiseq tail
**Oligos for nested PCR**		
I7-B01	CAG AAG ACG GCA TAC GAG ATC GTG ATG TGA CTG GAG TTC AGA CGT GTG C	Hiseq tail mutiplex
I7-B02	CAG AAG ACG GCA TAC GAG ATA CAT CGG TGA CTG GAG TTC AGA CGT GTG C	Hiseq tail mutiplex
I7-B03	CAG AAG ACG GCA TAC GAG ATG CCT AAG TGA CTG GAG TTC AGA CGT GTG C	Hiseq tail mutiplex
I7-B04	CAG AAG ACG GCA TAC GAG ATT GGT CAG TGA CTG GAG TTC AGA CGT GTG C	Hiseq tail mutiplex
I7-B05	CAG AAG ACG GCA TAC GAG ATC ACT GTG TGA CTG GAG TTC AGA CGT GTG C	Hiseq tail mutiplex
I7-B06	CAG AAG ACG GCA TAC GAG ATA TTG GCG TGA CTG GAG TTC AGA CGT GTG C	Hiseq tail mutiplex
I7-B07	CAG AAG ACG GCA TAC GAG ATG ATC TGG TGA CTG GAG TTC AGA CGT GTG C	Hiseq tail mutiplex
I7-B08	CAG AAG ACG GCA TAC GAG ATT CAA GTG TGA CTG GAG TTC AGA CGT GTG C	Hiseq tail mutiplex
I7-B09	CAG AAG ACG GCA TAC GAG ATC TGA TCG TGA CTG GAG TTC AGA CGT GTG C	Hiseq tail mutiplex
I7-B10	CAG AAG ACG GCA TAC GAG ATA AGC TAG TGA CTG GAG TTC AGA CGT GTG C	Hiseq tail mutiplex
I7-B11	CAG AAG ACG GCA TAC GAG ATG TAG CCG TGA CTG GAG TTC AGA CGT GTG C	Hiseq tail mutiplex
I7-B12	CAG AAG ACG GCA TAC GAG ATT ACA AGG TGA CTG GAG TTC AGA CGT GTG C	Hiseq tail mutiplex
I7-R01	CAG AAG ACG GCA TAC GAG ATT AGT GCG TGA CTG GAG TTC AGA CGT GTG C	Hiseq tail mutiplex
I7-R02	CAG AAG ACG GCA TAC GAG ATG CTA CAG TGA CTG GAG TTC AGA CGT GTG C	Hiseq tail mutiplex
I7-R03	CAG AAG ACG GCA TAC GAG ATA ATC CGG TGA CTG GAG TTC AGA CGT GTG C	Hiseq tail mutiplex

I7-R04	CAG AAG ACG GCA TAC GAG ATA CTG GTG TGA CTG GAG TTC AGA CGT GTG C	Hiseq tail mutiplex
I7-R05	CAG AAG ACG GCA TAC GAG ATT GTC ACG TGA CTG GAG TTC AGA CGT GTG C	Hiseq tail mutiplex
I7-R06	CAG AAG ACG GCA TAC GAG ATC GGT TAG TGA CTG GAG TTC AGA CGT GTG C	Hiseq tail mutiplex
I7-R07	CAG AAG ACG GCA TAC GAG ATG TCT AGG TGA CTG GAG TTC AGA CGT GTG C	Hiseq tail mutiplex
I7-R08	CAG AAG ACG GCA TAC GAG ATT GAA CTG TGA CTG GAG TTC AGA CGT GTG C	Hiseq tail mutiplex
I7-R09	CAG AAG ACG GCA TAC GAG ATC TAG TCG TGA CTG GAG TTC AGA CGT GTG C	Hiseq tail mutiplex
I7-R10	CAG AAG ACG GCA TAC GAG ATA TCG AAG TGA CTG GAG TTC AGA CGT GTG C	Hiseq tail mutiplex
I7-R11	CAG AAG ACG GCA TAC GAG ATC CGA TGG TGA CTG GAG TTC AGA CGT GTG C	Hiseq tail mutiplex
I7-R12	CAG AAG ACG GCA TAC GAG ATG AAC ATG TGA CTG GAG TTC AGA CGT GTG C	Hiseq tail mutiplex
**oligos for RAG1A/B locus**		
bioRAG1A/B	Biotin/GGA CTG CTG GAG ATT GCT CCA GAG AGG GTT TCC CCT CAA AGG AAT CCT TC	primer extension
I5-R1-RAG1A/B	ACT CTT TCC CTA CAC GAC GCT CTT CCG ATC TAG CGA GGA TCT CAC CCG GAA CAG C	Hiseq tail mutiplex
I5-R2-RAG1A/B	ACT CTT TCC CTA CAC GAC GCT CTT CCG ATC TGC CTA GGA TCT CAC CCG GAA CAG C	Hiseq tail mutiplex
I5-R3-RAG1A/B	ACT TTT CCC TAC ACG ACG CTC TTC CGA TCT AGG AAG GAT CTC ACC CGG AAC AGC	Hiseq tail mutiplex
I5-R4-RAG1A/B	ACT CTT TCC CTA CAC GAC GCT CTT CCG ATC TTC AGA GGA TCT CAC CCG GAA CAG C	Hiseq tail mutiplex
RAG1A-RFLP-F	GGG AGG CAA AGA TGA ATC AAA G	RAG1A-RFLP
RAG1A-T7EI-F	CCC CCT GGA AGA CTG CTT TA	RAG1A-RFLP
RAG1A-R/T-R	AGG ACT GCT GGA GAT TGC TC	RAG1A-RFLP/T7EI
**oligos for RAG1C locus**		
bioRAG1C	Biotin/CCT GAG AAC AAT GAA AAC AAG TC	primer extension
I5-R1-RAG1C	ACT CTT TCC CTA CAC GAC GCT CTT CCG ATC TAG CGT ATC AAT ATC CCA CTG ATG TAT C	Hiseq tail mutiplex
I5-R2-RAG1C	ACT CTT TCC CTA CAC GAC GCT CTT CCG ATC TGC CTT ATC AAT ATC CCA CTG ATG TAT C	Hiseq tail mutiplex
I5-R3-RAG1C	ACT CTT TCC CTA CAC GAC GCT CTT CCG ATC TAG GAT ATC AAT ATC CCA CTG ATG TAT C	Hiseq tail mutiplex
I5-R4-RAG1C	ACT CTT TCC CTA CAC GAC GCT CTT CCG ATC TTC AGT ATC AAT ATC CCA CTG ATG TAT C	Hiseq tail mutiplex

**oligos for MYC locus1**		
bioMYC1	Biotin/CGG GAA GGA AGT TGG CAT CTG TCC TG	primer extension
I5-R1-MYC1	ACT CTT TCC CTA CAC GAC GCT CTT CCG ATC TAG CGA GAG CCA TTC TCT GGC TCA G	Hiseq tail mutiplex
I5-R2-MYC1	ACT CTT TCC CTA CAC GAC GCT CTT CCG ATC TGC CTA GAG CCA TTC TCT GGC TCA G	Hiseq tail mutiplex
I5-R3-MYC1	ACT CTT TCC CTA CAC GAC GCT CTT CCG ATC TAG GAA GAG CCA TTC TCT GGC TCA G	Hiseq tail mutiplex
I5-R4-MYC1	ACT CTT TCC CTA CAC GAC GCT CTT CCG ATC TTC AGA GAG CCA TTC TCT GGC TCA G	Hiseq tail mutiplex
**oligos for MYC locus2**		
bioMYC2	Biotin/TTG CGA CTC TCA GCT GAA TCC ACT GCT G	primer extension
I5-R1-MYC2	ACT CTT TCC CTA CAC GAC GCT CTT CCG ATC TAG CGT GCT CCC ATG GCA TCA TGA C	Hiseq tail mutiplex
I5-R2-MYC2	ACT CTT TCC CTA CAC GAC GCT CTT CCG ATC TGC CTT GCT CCC ATG GCA TCA TGA C	Hiseq tail mutiplex
I5-R3-MYC2	ACT CTT TCC CTA CAC GAC GCT CTT CCG ATC TAG GAT GCT CCC ATG GCA TCA TGA C	Hiseq tail mutiplex
I5-R4-MYC2	ACT CTT TCC CTA CAC GAC GCT CTT CCG ATC TTC AGT GCT CCC ATG GCA TCA TGA C	Hiseq tail mutiplex
**oligos for EMX1 locus**		
biotinEMX1	Biotin/CCC ATC AGG CTC TCA GCT CAG CCT GAG TGT TGA G	primer extension
I5-R1-EMX1	ACT CTT TCC CTA CAC GAC GCT CTT CCG ATC TAG CGC CCA GGT GAA GGT GTG GTT C	Hiseq tail mutiplex
I5-R2-EMX1	ACT CTT TCC CTA CAC GAC GCT CTT CCG ATC TGC CTC CCA GGT GAA GGT GTG GTT C	Hiseq tail mutiplex
I5-R3-EMX1	ACT CTT TCC CTA CAC GAC GCT CTT CCG ATC TAG GAC CCA GGT GAA GGT GTG GTT C	Hiseq tail mutiplex
I5-R4-EMX1	ACT CTT TCC CTA CAC GAC GCT CTT CCG ATC TTC AGC CCA GGT GAA GGT GTG GTT C	Hiseq tail mutiplex
**oligos for RAG1A off-targets**		
bio*Sp*CAS9-RAG1A-OT6	GGG TGT CAC GGG CCG TGG GAC AGG TC	primer extension
I5-R1-*Sp*CAS9-RAG1A-OT6	ACT CTT TCC CTA CAC GAC GCT CTT CCG ATC TAG CGC ATC GGT TGC CAT GGC TAC TG	Hiseq tail mutiplex
I5-R2-*Sp*CAS9-RAG1A-OT6	ACT CTT TCC CTA CAC GAC GCT CTT CCG ATC TGC CTC ATC GGT TGC CAT GGC TAC TG	Hiseq tail mutiplex
I5-R3-*Sp*CAS9-RAG1A-OT6	ACT CTT TCC CTA CAC GAC GCT CTT CCG ATC TAG GAC ATC GGT TGC CAT GGC TAC TG	Hiseq tail mutiplex
bio*Sp*CAS9-RAG1A-OT8	CCT CCC TGA CTA GTG GTT AAC GGA CTT G	primer extension

I5-R1-*Sp*CAS9-RAG1A-OT8	ACT CTT TCC CTA CAC GAC GCT CTT CCG ATC TAG CGC GCT TTC CTG GCG GTT ACA G	Hiseq tail mutiplex
I5-R2-*Sp*CAS9-RAG1A-OT8	ACT CTT TCC CTA CAC GAC GCT CTT CCG ATC TGC CTC GCT TTC CTG GCG GTT ACA G	Hiseq tail mutiplex
I5-R3-*Sp*CAS9-RAG1A-OT8	ACT CTT TCC CTA CAC GAC GCT CTT CCG ATC TAG GAC GCT TTC CTG GCG GTT ACA G	Hiseq tail mutiplex
RAG1A-OT1-F	GGC TGT CCA TGT TGT CCT TC	*In vitro* digestion
RAG1A-OT1-R	ACA CCT CTG GTT AGA GAT AAA GTG	*In vitro* digestion
RAG1A-OT2-F	CTT GCG ATC TGT CTG TCT CC	*In vitro* digestion
RAG1A-OT2-R	GAT GTG GAG ACT GAT TAA GCC	*In vitro* digestion
RAG1A-OT3-F	AGG CCA GTG GTC ACG TGG CTC	*In vitro* digestion
RAG1A-OT3-R	GGC CGA GGC CTG GAG ACA GG	*In vitro* digestion
RAG1A-OT4-F	ACA GAC ATA CAC GTG AGG TC	*In vitro* digestion
RAG1A-OT4-R	AAG TAC AGT CGG ACT TGG CAG	*In vitro* digestion
RAG1A-OT5-F	GCT GCC TCA GCG GCC AGG GCC	*In vitro* digestion
RAG1A-OT5-R	TCC TCC CAT CCC AAG GTG CC	*In vitro* digestion
RAG1A-OT6-F	GGC CTG ATG GCC AGA GAG GG	*In vitro* digestion
RAG1A-OT6-R	GAA TAA TTA CAG GGG AGT CAG CC	*In vitro* digestion
RAG1A-OT7-F	CAT GAG CCT CTG GAA TCT GTC	*In vitro* digestion
RAG1A-OT7-R	GTC ACA TTC ACA GGT CCG AG	*In vitro* digestion
RAG1A-OT8-F	TCT GGC TAA GCC GTT AGG CGC	*In vitro* digestion
RAG1A-OT8-R	CCT GTG TTT TCT GGA CAC AGA AC	*In vitro* digestion
RAG1A-on-target-F	GGG AGG CAA AGA TGA ATC AAA G	*In vitro* digestion
RAG1A-on-target-R	GGA TCG AGT CAA CAT CTG CC	*In vitro* digestion
Negative-control-F	GCC TCT GAA AAC CTA TGG CC	*In vitro* digestion
Negative-control-R	GTT GCA CAA TGG CAG AGA CC	*In vitro* digestion
